# Transcription-driven genome organization: a model for chromosome structure and the regulation of gene expression tested through simulations

**DOI:** 10.1093/nar/gky763

**Published:** 2018-09-18

**Authors:** Peter R Cook, Davide Marenduzzo

**Affiliations:** 1 Sir William Dunn School of Pathology, University of Oxford, South Parks Road, Oxford OX1 3RE, UK; 2 SUPA, School of Physics, University of Edinburgh, Peter Guthrie Tait Road, Edinburgh, EH9 3FD, UK

## Abstract

Current models for the folding of the human genome see a hierarchy stretching down from chromosome territories, through A/B compartments and topologically-associating domains (TADs), to contact domains stabilized by cohesin and CTCF. However, molecular mechanisms underlying this folding, and the way folding affects transcriptional activity, remain obscure. Here we review physical principles driving proteins bound to long polymers into clusters surrounded by loops, and present a parsimonious yet comprehensive model for the way the organization determines function. We argue that clusters of active RNA polymerases and their transcription factors are major architectural features; then, contact domains, TADs and compartments just reflect one or more loops and clusters. We suggest tethering a gene close to a cluster containing appropriate factors—a transcription factory—increases the firing frequency, and offer solutions to many current puzzles concerning the actions of enhancers, super-enhancers, boundaries and eQTLs (expression quantitative trait loci). As a result, the activity of any gene is directly influenced by the activity of other transcription units around it in 3D space, and this is supported by Brownian-dynamics simulations of transcription factors binding to cognate sites on long polymers.

## INTRODUCTION

Current reviews of DNA folding in interphase human nuclei focus on levels in the hierarchy between looped nucleosomal fibers and chromosome territories (1,2). Hi-C—a high-throughput variant of chromosome conformation capture (3C)—provides much of our knowledge in this area. The first Hi-C maps had low resolution (∼1 Mb), and revealed plaid-like patterns of A (active) and B (inactive) compartments that often contact others of the same type (3). Higher-resolution (∼40 kb) uncovered topologically-associating domains (TADs); intra-TAD contacts were more frequent than inter-TAD ones (4,5). Still higher-resolution (∼1 kbp) gave contact loops delimited by cohesin and CTCF bound to cognate motifs in convergent orientations (6), as well as domains not associated with CTCF, called ‘ordinary’ or ‘compartmental’ domains (6,7). [Nomenclature can be confusing, as domains of different types are generally defined using different algorithms.]

Despite these advances, critical features of the organization remain obscure. For example, Hi-C still has insufficient resolution to detect many loops seen earlier (Supplementary Note 1). Moreover, most mouse domains defined using the Arrowhead algorithm persist when CTCF is degraded (8) (see also bioRxiv: https://doi.org/10.1101/118737). and many other organisms get by without the protein, (e.g. *Caenorhabditis elegans* (9), *Neurospora* (10), budding (11) and fission yeast (12), *Arabidopsis thaliana* (13), and *Caulobacter crescentus* (14)). Therefore, it seems likely that loops stabilized by CTCF are a recent arrival in evolutionary history.

The relationship between structure and function is also obscure (15). For example, cohesin—which is a member of a conserved family—plays an important structural role in stabilizing CTCF loops (Supplementary Note 2), but only a minor functional role in human gene regulation as its degradation affects levels of nascent messenger RNAs (mRNAs) encoded by only 64 genes (16). Widespread use of vague terms like ‘regulatory neighborhood’ and ‘context’ reflects this deficit in understanding. Here, we discuss physical principles constraining the system, and describe a parsimonious model where clusters of active RNA polymerases and its transcription factors are major structural organizers—with contact domains, TADs, and compartments just reflecting this underlying framework. This model naturally explains how genes are regulated, and provides solutions to many current puzzles.

## SOME PHYSICAL PRINCIPLES

### Chromatin mobility

Time-lapse imaging of a GFP-tagged gene in a living mammalian cell is consistent with it diffusing for ∼1 min through a ‘corral’ in chromatin, ‘jumping’ to a nearby corral the next and bouncing back to the original one (17). Consequently, a gene explores a volume with a diameter of ∼250 nm in a min, ∼750 nm in 1 h and ∼1.4 μm in 24 h (18); therefore, it inspects only part of one territory in ∼24 h, as a yeast gene—which diffuses as fast—ranges throughout its smaller nucleus.

### Entropic forces

Monte Carlo simulations of polymers confined in a sphere uncovered several entropic effects depending solely on excluded volume (19,20). Flexible thin polymers (‘euchromatin’) spontaneously move to the interior, and stiff thick ones (‘heterochromatin’) to the periphery—as seen in human nuclei (Supplementary Figure S1Ai); ‘euchromatin’ loses more configurations (and so entropy) than ‘heterochromatin’ when squashed against the lamina, and so ends up internally. Stiff polymers also contact each other more than flexible ones; this favors phase separation and formation of distinct A and B compartments. Additionally, linear polymers intermingle, but looped ones segregate into discrete territories (Supplementary Figure S1Aii).

### Ellipsoidal territories and *trans* contacts

Whether a typical human gene diffuses within its own territory and makes *cis* contacts (i.e. involving contacts with the same chromosome), or visits others to make *trans* ones depends significantly on territory shape. Children who buy M$\&$Ms and Smarties sense ellipsoids pack more tightly than spheres of similar volume; packed ellipsoids also touch more neighbours than spheres (Supplementary Figure S1B). As territories found in cells and simulations are ellipsoidal, and as much of the volume of ellipsoids is near the surface, genes should make many *cis* contacts plus some *trans* ones (Supplementary Figure S1).

### Some processes driving looping

If human chromosomes were a polymer melt in a sphere, two loci 40 Mb distant on the genetic map would be ∼4 μm apart in 3D space and interact as infrequently as loci on different chromosomes. If the two were 10, 1 or 0.1 Mb apart, they would interact with probabilities of ∼2 × 10^−5^, ∼5 × 10^−4^ and ∼1.5 × 10^−2^, respectively (calculated using a 20 nm fiber, 50 bp/nm and a threshold of 50 nm for contact detection; see also (1)). Hi-C shows some contacts occur more frequently; this begs the question—what drives looping?

**Figure 1.**
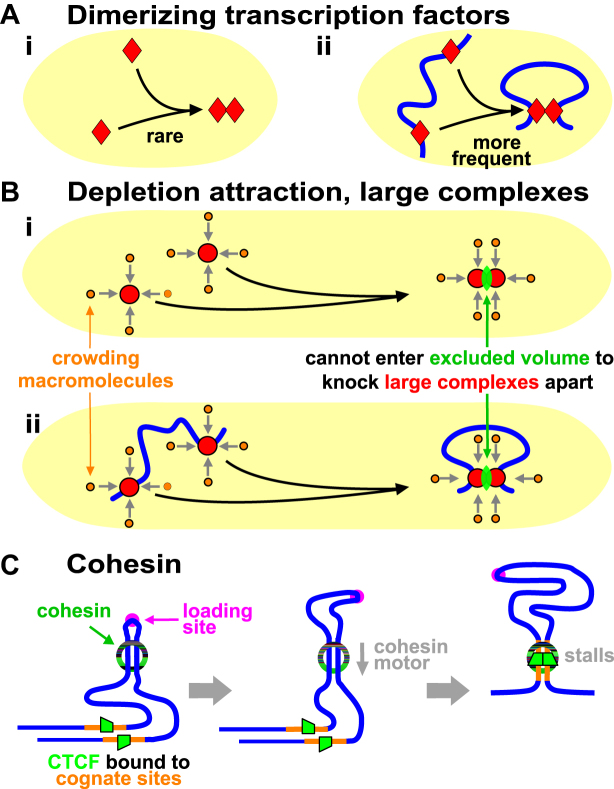
Some drivers of looping. (**A**) Dimerizing factors (equilibrium constant ∼10^−7^ M). (**i**) If present at a typical concentration (∼1 nM), <1% factors dimerize. (**ii**) Binding to cognate sites 10 kbp apart on DNA increases local concentrations, and ∼67% are now dimers stabilizing loops (21). (**B**) The depletion attraction. (**i**) In crowded nuclei, small brown molecules (diameter <5 nm) bombard (grey arrows) larger red complexes (5–25 nm). If large complexes collide, smaller molecules are sterically excluded from the green volume between the two and cannot knock them apart; consequently, small molecules exert a force on opposite sides of larger complexes keeping them together. (**ii**) If large complexes are bound to DNA, this force stabilizes a loop. (**C**) Cohesin. After loading, a cohesin ring embraces two fibers to stabilize a mini loop; this loop enlarges as the ring uses an inbuilt motor to move down the fiber until stalled by CTCF bound to convergent sites.

One process is the classical one involving promoter-enhancer contacts (21). We discuss later that contacting partners are often transcriptionally active. We also use the term ‘promoter’ to describe the 5′ end of both genic and non-genic units, and ‘factor’ to include both activators and repressors. Many factors (often bound to polymerases) can bind to DNA and each other (e.g. YY1 (22)). Binding to two cognate sites spaced 10 kb apart creates a high local concentration, and—when two bound factors collide—dimerization stabilizes a loop if entropic looping costs are not prohibitive (Figure 1A). Such loops persist as long as factors remain bound (typically ∼10 s).

Another mechanism—the ‘depletion attraction’—is non-specific. It originates from the increase in entropy of macromolecules in a crowded cell when large complexes come together (Figure 1Bi (23)). Modeling indicates this attraction can cluster bound polymerases and stabilize loops (Figure 1Bii) that persist for as long as polymerases remain bound (i.e. seconds to hours; below).

A third mechanism involves cohesin—a ring-like complex that clips on to a fiber like a carabiner on a climber’s rope. In Hi-C maps, many human domains are contained in loops apparently delimited by CTCF bound to cognate sites in convergent orientations (6). Such ‘contact loops’—many with contour lengths of >1 Mb—are thought to arise as follows. A cohesin ring binds at a ‘loading site’ to form a tiny loop, this loop enlarges as an in-built motor translocates the ring down the fiber, and enlargement ceases when CTCF bound to convergent sites blocks further extrusion (Figure 1C (24,25)). This is known as the ‘loop-extrusion model’. We note that other mechanisms could enlarge such loops (including one not involving a motor; Supplementary Note 2), and that loop extrusion (by whatever mechanism) and its blocking by convergent CTCF sites can be readily incorporated into the model that follows.

### A transcription-factor model

We now review results of simulations involving what we will call the ‘transcription-factor model’. This incorporates the few assumptions implicit in the classical model illustrated in Figure 1A: spheres (‘factors’) bind to selected beads in a string (‘cognate sites’ on ‘chromatin fibers’) to form molecular bridges stabilizing loops (26–30). This superficially simple model yields several unexpected results.

**Figure 2.**
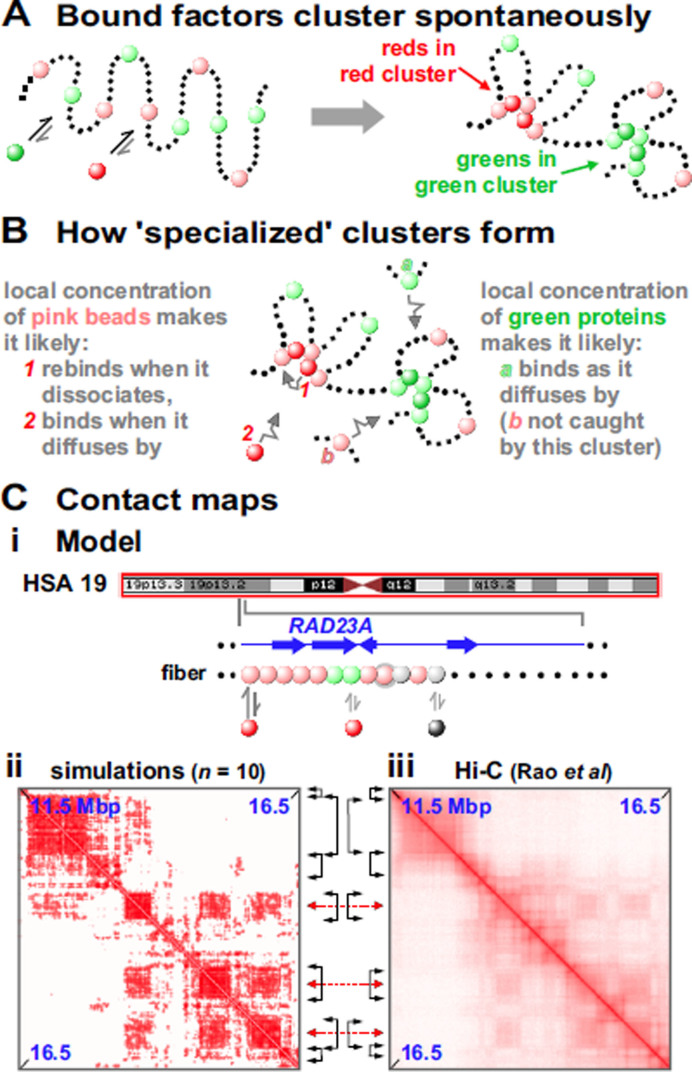
A process driving the spontaneous clustering of multivalent factors (a.k.a., the ‘bridging-induced attraction’). (**A**) Overview of one Brownian-dynamics simulation. Red and green ‘factors’ (colored spheres) bind reversibly to ‘chromatin’ (a string of beads); red factors bind only to pink beads, green factors only to light-green ones (non-binding beads shown as black dots). Bound factors spontaneously cluster—red with red and green with green—despite any specified interactions between proteins or between beads. (**B**) Explanation. Local concentrations create positive-feedback loops driving growth of nascent clusters; bound factors and binding beads rarely escape, and additional factors/beads are caught as they diffuse by. Red and green clusters are inevitably separate in 3D space because their cognate binding sites are separate in 1D sequence space. Cluster growth is limited by entropic costs of crowding together ever-more loops. (**C**) Comparison of contact maps obtained from 10 simulations (28) and Hi-C (6). (**i**) The model. The whole of chromosome 19 (red box) in GM12878 cells was simulated, and the zoom shows the region around *RAD23A*, which is active in these cells. Each bead in the fiber is colored according to whether the corresponding region is transcriptionally highly active (pink), weakly active (green) or silent (grey) on the Broad ChromHMM track on the UCSC browser; one bead carries both active and silent marks and so bears two colors. Pink (activating) and black (repressing) factors bind to cognate beads as indicated (the doubly-colored bead binds both factors); all other beads (black dots) are non-binding. (**ii, iii**) Contact maps are similar. Black double-headed arrows: limits of prominent TADs on diagonal. Red double-headed arrows: centers of off-diagonal blocks marking compartments.

First, and extraordinarily, bound factors cluster spontaneously in the absence of any specified DNA–DNA or protein–protein interactions (Figure 2A (27)). This clustering requires bi- or multi-valency (so factors can bridge different regions and make loops) plus reversible binding (otherwise the system does not evolve), and it occurs robustly with respect to changes in DNA–protein affinity and factor number. The process driving it was dubbed the ‘bridging-induced attraction’ (27). We stress this attraction occurs spontaneously without the need to specify any additional forces between one bead and another, or between one protein and another.

The basic mechanism yielding clustering is a simple positive feedback loop which works as sketched in Figure 2A and B. First, proteins bind to chromatin (Figure 2A). Then, once a bridge forms, the local density of binding sites (e.g. pink spheres in Figure 2A) inevitably increases. This attracts further factors from the soluble pool (like *2* in Figure 2B): their binding further increases the local chromatin concentration (through bridging) creating a virtuous cycle which repeats. This triggers the self-assembly of stable protein clusters, where growth is eventually limited by entropic crowding costs (28). Several factors cluster in nuclei (e.g. Sox2 in living mouse cells (31)) and the bridging-induced attraction provides a simple and general explanation for this phenomenon.

This process drives local phase separation of polymerases and factors, and so naturally explains how super-enhancer (SE) clusters form (Supplementary Figure S2Ai (32)). This generic tendency to cluster will be augmented by specific protein–protein and DNA–protein interactions, with their balance determining whether protein or DNA lies at the core. Similarly, the same process—this time augmented by HP1, a multivalent protein that staples together histones carrying certain modifications—could drive phase separation and compaction of inactive heterochromatin (Supplementary Figure S2Aii (33,34)).

### Creating stable clusters of different types, TADs and compartments

This transcription-factor model yields a second remarkable result: red and green factors binding to distinct sites on the string self-assemble into distinct clusters containing only red factors or only green ones (Figure 2A (28)). This has a simple basis: the model specifies that red and green binding sites are separate in 1D sequence space (as they are *in vivo*), so they are inevitably in different places in 3D space (Figure 2B).

A third result is that clusters and loops self-assemble into ‘TADs’ and ‘A/B compartments’ (26–28). Thus, if chromosome 19 in human GM12878 cells is modeled as a string of beads colored according to whether corresponding regions are active or inactive, binding of just red and black spheres (‘activators’ and ‘repressors’) yields contact maps much like Hi-C ones (Figure 2C). As neither TADs, compartments, nor experimental Hi-C data are used as inputs, this points to polymerases and their factors driving the organization without the need to invoke roles for higher-order features (see also (7)). We suggest TADs arise solely by aggregation of pre-existing loops/clusters (note that degradation of cohesin or its loader induces TAD disappearance and the emergence of complex sub-structures, as A/B compartments persist and become more prominent (16,35)).

The simple transcription-factor model has been extended to explain how pre-existing red clusters can evolve into green clusters, or persist for hours as individual factors exchange with the soluble pool in seconds—as in photo-bleaching experiments (Supplementary Figure S3A,B (28,36)). Additionally, introducing ‘bookmarking’ factors that bind selected beads (genomic sequences), as well as ‘writers’ that ‘mark’ chromatin beads and ‘readers’ which bind beads with specific marks, can create local ‘epigenetic states’ and epigenetic domains (e.g. domains of red and green marks, representing for instance active or inactive histone modifications). Such domains spontaneously establish around bookmarks, and are stably inherited through ‘semi-conservative replication’, when half of the marks are erased (and/or some of the bookmarks are lost due to dilution (37,38); Supplementary Figure S3C).

## A PARSIMONIOUS MODEL: CLUSTERS OF POLYMERASES AND FACTORS

**Figure 3.**
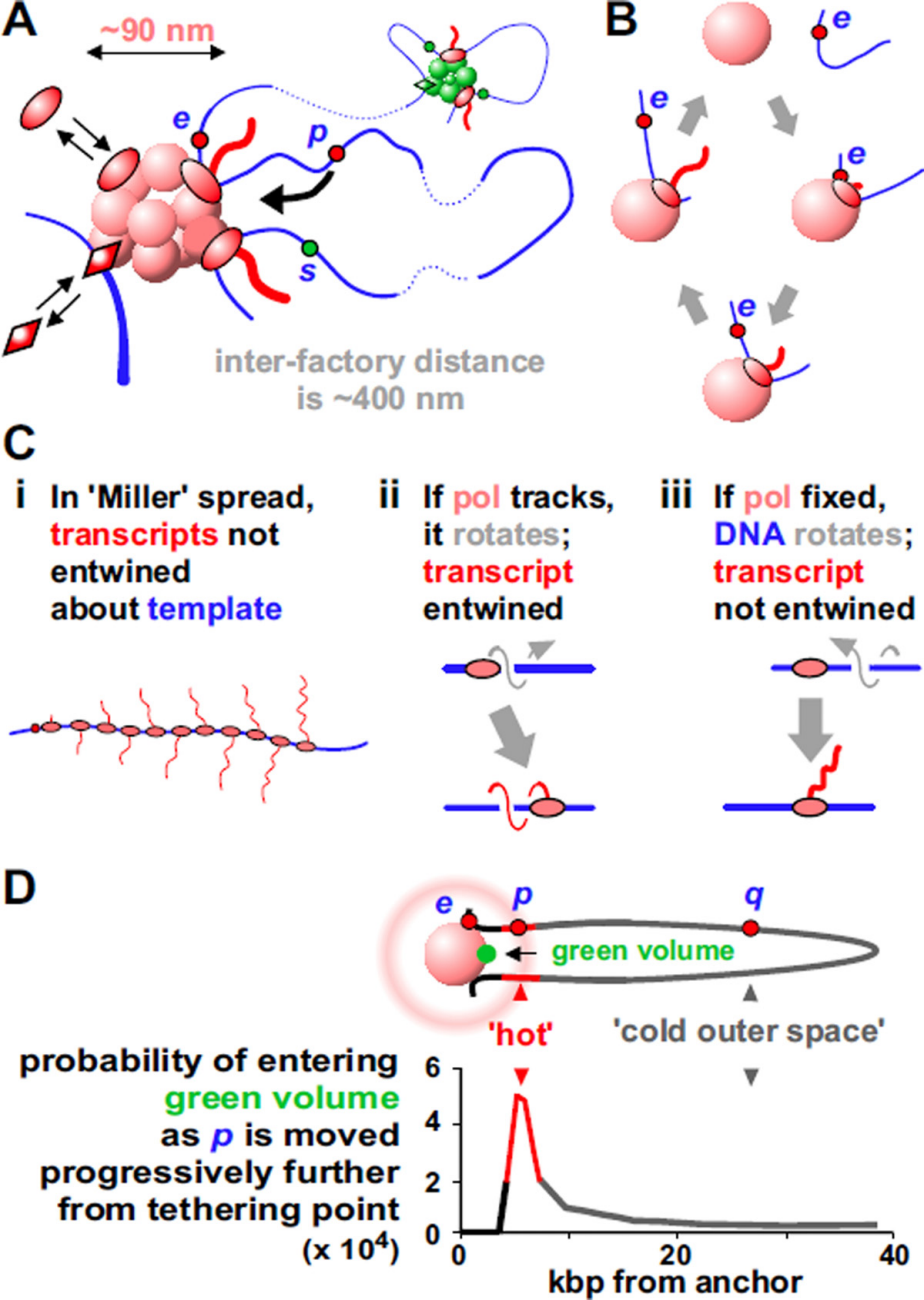
Transcription factories in human cells. (**A**) Clusters organize loops stabilized by polymerases (ovals) and factors (lozenges). There are ∼16 loops per factory, but only a few are shown here and subsequently. Red and green factories specialize in transcribing different gene sets. Promoters tend to be transcribed in factories of the same color (because they are rich in appropriate factors); here, *p* and *s* can often visit the pink factory, but only *p* is likely to initiate there. (**B**) A transcription cycle. Promoter *e* collides with a polymerase in the factory (shown as a solid sphere from now on), initiates, and the fixed polymerase reels in the template as it extrudes a transcript; the template detaches on termination. (**C**) ‘Miller’ spreads. (**i**) A Christmas tree. (**ii**) If the polymerase tracks, it rotates about the template once for every 10-bp transcribed to give an entwined transcript. (**iii**) If immobile, the template rotates and the transcript is not entwined. Topoisomerases remove twin domains of supercoiling in both (ii) and (iii) (41). (**D**) Tether length affects how often a promoter visits a factory. Top: a 77-kbp loop tethered to a 75-nm sphere; intuition suggests *p* visits the green volume more than *q*. Bottom: results of Monte-Carlo simulations confirm this intuition. Adapted from (42) with permission; copyright 2006 Elsevier.

These physical principles lead naturally to a model in which a central architectural feature is a cluster of active polymerases/factors surrounded by loops—a ‘transcription factory’. A factory was defined as a site containing ≥2 polymerases active on ≥2 templates, just to distinguish it from cases where two enzymes are active on one (Figure 3A (39,40)). Much as car factories contain high local concentrations of parts required to make cars efficiently, these factories contain machinery that acts through the law of mass action to drive efficient RNA production. For RNA polymerase II in HeLa, the concentration in a factory (i.e. ∼1 mM) is ∼1000-fold higher than the soluble pool; consequently, essentially all transcription occurs in factories (Supplementary Note 3; Supplementary Note 4 describes some properties of factories).

In all models, a gene only becomes active if appropriate polymerases (i.e. I, II or III) and factors are present; in this one, there are three more requirements. First, active polymerases are transiently immobile when active; they reel in their templates as they extrude their transcripts (Figure 3B). This contrasts with the traditional view where they track like locomotives down templates. Arguably, the best (perhaps only) evidence supporting the traditional view comes from iconic images of ‘Christmas trees’; a 3D structure is spread in 2D, and imaged in an electron microscope—polymerases are caught in the act of making RNA (Figure 3Ci). However, polymerases moving along helical templates generate entwined transcripts (Figure 3Cii), but these transcripts appear as un-entwined ‘branches’ in ‘Christmas trees’. How could such structures arise? As transcription requires lateral and rotational movement along/around the helix, we suggest templates move (not polymerases) to give un-entwined transcripts (Figure 3Ciii). Consequently, these images provide strong evidence against the traditional model, not for it (see also Supplementary Note 5, Supplementary Figure S4).

Second, in order to initiate, a promoter must have a high probability of colliding with a polymerase, and—as the highest polymerase concentrations are found in/around factories—this means the enzyme must first diffuse into/near a factory. [We remain agnostic as to the order with which promoter, polymerase, factors and factory bind to each other, and note that the participants in nucleotide excision repair—a process arguably better understood than transcription (43)—are not assembled one after the other; instead the productive complex forms once all participants happen to collide simultaneously into each other.] In Figure 3D, intuition suggests *p* often visits the nearby green volume, whereas *q* mainly roams ‘outer space’; simulations and experiments confirm this (42,44). Consequently, active genes tend to be tethered close to a factory, and inactive genes further away. Promoter-factory distances also seem to remain constant as nuclear volume changes; when mouse ES cells differentiate and their nuclei become 2-fold larger or 2-fold smaller, experiments show the system spontaneously adapts to ensure these distances remain roughly constant, and new simulations confirm this (Supplementary Figure S6; Supplementary Note 6).

Third, there are different types of factory (red and green clusters in Figure 3A), and a gene must visit an appropriate one to initiate. Just as some car factories make Toyotas and others Teslas, different factories specialize in transcribing different sets of genes. For example, distinct ‘ERα’, ‘KLF1’ and ‘NFκB’ factories specialize in transcribing genes involved in the estrogen response, globin production, and inflammation, respectively (45–47).

These three principles combine to ensure the structure is probabilistic and dynamic, with current shape depending on past and present environments. For example, as *e* in Figure 3D is transcribed, loop length changes continuously. And when *e* terminates, it dissociates; then, its diffusional path may take it back to the same factory where it may (or may not) re-initiate to reform a loop. Alternatively, *e* may spend some time diffusing through outer space before rebinding to the same or a different factory. Consequently, as factors and polymerase bind and dissociate, factories morph, loops appear and disappear—and the looping pattern of every chromosomal segment changes from moment to moment. Then, it is unlikely the 3D structure of any chromosome is like that of its homolog, either in the same cell or any other cell in a clonal population.

These physical principles also lead naturally to an explanation of how genes become inactive. Thus, *q* in Figure 3D is inactive because it lies far away from an appropriate factory and is unlikely to collide with a polymerase there. We speculate that inactivity results in histone modifications that thicken the fiber, so entropic effects collapse it with other heterochromatic fibers into B compartments and the nuclear periphery (as in Supplementary Figure S1Ai).

## SOME DIFFICULT-TO-EXPLAIN OBSERVATIONS

We now describe results easily explained by this model, but difficult or impossible to explain by others without additional complicated assumptions (see also Supplementary Note 7).

### Most contacts are between active transcription units

Contacts seen by 3C-based approaches often involve active promoters and enhancers; for example, FIRES (frequently-interacting regions) in 14 different human tissues and 7 human cell lines are usually active enhancers (48). Similarly, contacts detected by an independent method—genome architecture mapping—again involve enhancers and/or genic transcription start/end sites (49). Why should active sequences lie together? As factories nucleate local concentrations of active units, we expect promoters and enhancers to dominate contact lists.

While 3C focuses on contacts between two DNA sequences, the ligation involved can join >2 together (24 is the current record), and these again generally encode active sequences (50,51). Why do so many active sequences contact each other? We expect to see co-ligations involving some/all of the many anchors in a typical factory.

Early studies also point to a correlation between transcription and structure. For example, switching on/off many mammalian genes correlates with their attachment/detachment (40). What underlies this? Our model requires that units must attach before they can be transcribed.

### Frequencies of *cis* and *trans* contacts

*Cis* Hi-C contacts fall off rapidly with increasing genetic distance, whereas *trans* ones are so rare they are often treated as background. However, ChIA-PET yields more *trans* than *cis* contacts when active sequences are selected by pulling down ERα or polymerase II (45,47). Our model again predicts this—active genes on different chromosomes are often co-transcribed in the same specialized factory (as genes diffuse out of one ellipsoidal territory into another).

In addition, *cis*:*trans* ratios can change rapidly, and we explain this by reference to ‘NFκB’ factories (47) (see also Supplementary Figure S5A). TNFα induces phosphorylation of NFκB, nuclear import of phospho-NFκB, and transcriptional initiation of many inflammatory genes including *SAMD4A*. Before induction, the *SAMD4A* promoter makes only a few local *cis* contacts (shown by 4C and ChIA-PET applied with a ‘pull-down’ of polymerase II); it spends most time roaming ‘outer space’ making a few chance contacts with nearby segments of its own loop, and—if it visits a factory—it cannot initiate in the absence of phospho-NFκB. But once phospho-NFκB appears (10 min after adding TNFα), it initiates. Then, NFκB binding sites in *SAMD4A* become tethered to the factory, these bind phospho-NFκB, exchange of the factor increases the local concentration, and this increases the chances that other inflammatory genes initiate when they pass by. And once they do, this creates a virtuous cycle; as more inflammatory genes initiate, more NFκB binding sites become tethered to the factory, the local NFκB concentration rises, this further increases the chances that passing responsive genes initiate, and the factory evolves into one specializing in transcribing inflammatory genes. As a result, the rapid concentration of inflammatory genes around the resulting ‘NFκB’ factory yields the rapid increase in *cis* and *trans* contacts between them seen by 3C-based methods and RNA-FISH (47).

### TADs exist at all scales

Intra- and inter-TAD contact frequencies differ only ∼2-fold; therefore, it is unsurprising that TAD calling depends on which algorithm is used, and the resolution achieved (52–55). However, it is surprising that TADs become more elusive as algorithms and resolution improve. For example, CaTCH (Caller of Topological Chromosomal Hierarchies) identifies a continuous spectrum of domains covering all scales; TADs do not stand out as distinct structures at any level in the hierarchy (55). Moreover, TADs are sometimes invisible in single-cell data (56,57), and—if detected—their borders weaken as cells progress through G1 into S phase (58). In our model, TADs do not exist as distinct entities representing anything other than one or more loops around one or more factories. [TADs are said to be major architectural features because they are invariant between cell types (4,5) and highly conserved (59). However, there are always slight differences between cell types that could reflect slight differences in expression profile, and the conservation could just reflect the conserved transcriptional pattern encoded by the underlying DNA sequence.]

### The relationship between TADs and transcription

Various studies address this issue, and give conflicting results. For example, in mouse neural progenitor cells, one of the two X chromosomes is moderately compacted and largely inactive. Inactive regions do not assemble into A/B compartments or TADs, unlike active ones. Moreover, in different clones, different regions in the inactive X escape inactivation, and these form TADs (60). Here, structure and activity are tightly correlated (in accord with our model). Similarly, inhibiting transcription in the fly leads to a general reorganization of TAD structure, and a weakening of border strength (61).

Another study points to some TADs appearing even though transcription is inhibited (62). After fertilization, the zygotic nucleus in the fly egg is transcriptionally inactive. As the embryo divides, zygotic genome activation occurs so that by nuclear cycle 8 (nc8), ∼180 genes are active, and these seem to nucleate a few TADs detected at nc12 (so transcriptional onset and the appearance of loops/TADs correlate—again in accord with our model). As more genes become active at nc13, 3-fold more TADs develop by nc14, and polymerase II plus Zelda (a zinc-finger transcription factor) are at boundaries (again a positive correlation). If transcriptional inhibitors are injected into embryos before nc8, boundaries and TADs seen at nc14 are less prominent, but some TADs still develop (implying loops/TADs appear independently of transcription, which is inconsistent with our model). However, interpretation is complicated. Although inhibitors reduce levels of five mRNAs already being expressed, they only slightly affect levels of polymerase II bound at the 5′ end of genes expressed at nc14; this indicates that inhibition is inefficient, so it remains possible that the remaining transcription stabilizes the loops/TADs seen.

Studies on mouse eggs and embryos also provide conflicting data. Thus, activity is lost as oocytes mature, and TADs plus A/B compartments disappear (56,63,64); therefore, loss of structure and activity again correlate (consistent with our model). After fertilization, the zygote contains two nuclei with different conformations; both contain TADs, but the maternal one lacks A/B compartments. Then, as transcription begins, TADs appear (again a positive correlation), but α-amanitin (a transcriptional inhibitor) does not prevent this (63,64)—which is inconsistent with our model. However, interpretation is again complicated: α-amanitin acts notoriously slowly (65), and inhibition was demonstrated indirectly (levels of steady-state poly(A)^+^ RNA fall, but reduction of intronic RNA would be a more direct indicator of inhibition).

Data from zebrafish make unified interpretation even more difficult. In contrast to some cases cited earlier, TADs and compartments exist before zygotic gene activation, and many of each are lost when transcription begins (66). Clearly, TAD-centric models will find it difficult to explain such conflicting data. In ours, TADs are not major architectural features determining function; they just reflect the underlying network of loops, and—even if all polymerases are inactive—bound factors can still stabilize some loops (and so TADs).

### Enhancers and super-enhancers

Enhancers are important regulatory motifs, but there remains little agreement on how they work (67). They were originally defined as motifs stimulating firing of genic promoters when inserted in either orientation upstream or downstream. However, their molecular marks are so like those of their targets (68) that FANTOM5 now defines them solely as promoters firing to yield eRNAs (enhancer RNAs) rather than mRNAs (69). Then, is it eRNA production or some role of the eRNA product that underlies function? Studies of the *Sfmbt2* enhancer in mouse ES cells indicates it is the former (70). Thus, deleting the eRNA promoter (but not downstream sequences) impairs enhancer activity; this points to the promoter being required. Moreover, inserting a poly(A) site just 40 bp down-stream of the eRNA promoter abolishes enhancer activity, and amounts of polymerase on the enhancer (and enhancer activity) increase as the insert is moved progressively 3′; this points to a reduction in transcription correlating with reduced enhancer activity.

**Figure 4.**
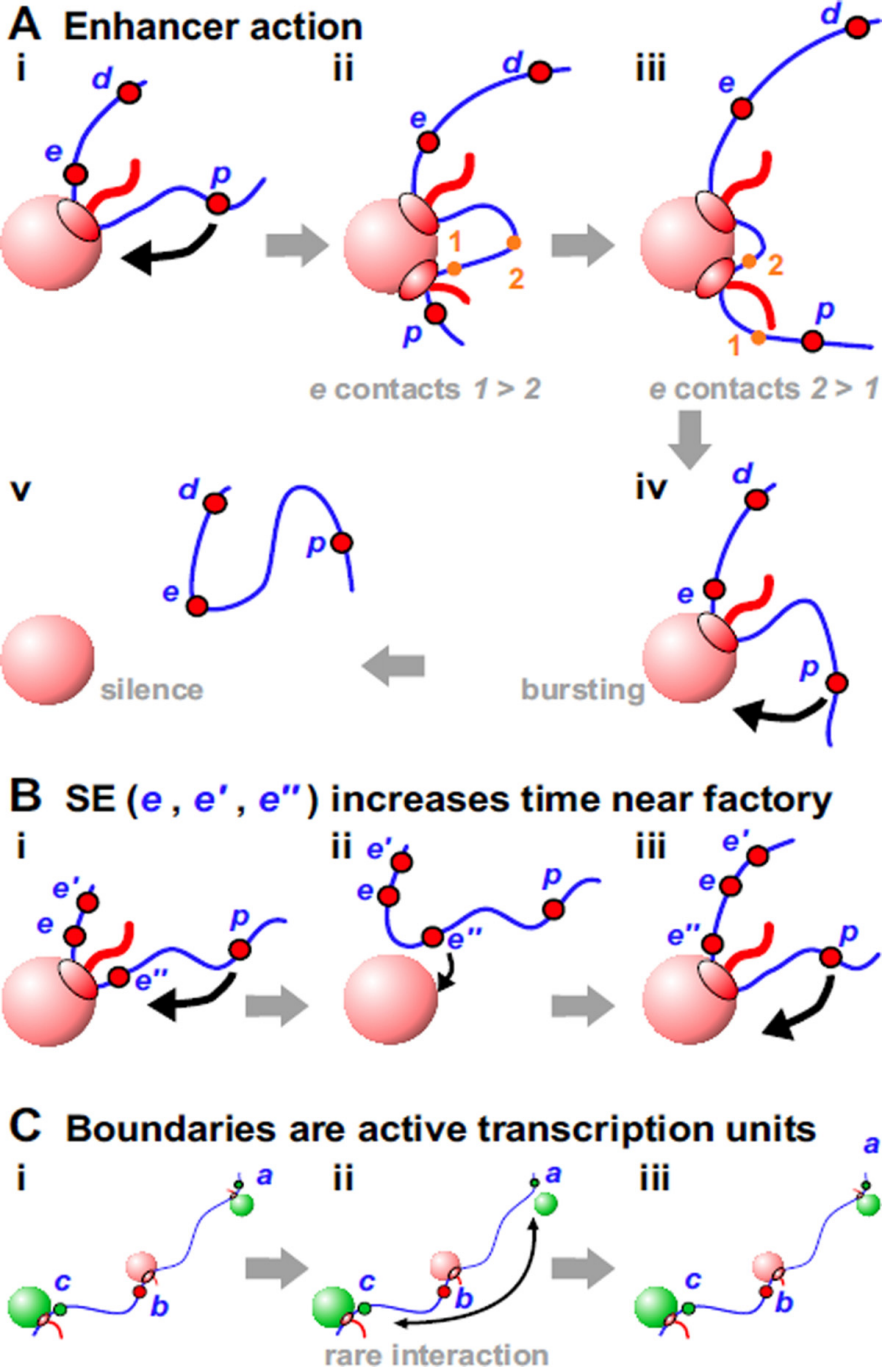
Enhancers and boundaries. (**A**) Enhancer action. (**i**) *p* is tethered by enhancer *e* close to a factory—so *p* is likely to collide with the factory. (**ii**) *p* has initiated, and the polymerase is about to transcribe *1*. (**iii**) The same polymerase will now transcribe *2*; then, *e-p* contacts apparently track with the polymerase away from *p*. (**iv**) Both polymerases now terminate, *e* and *p* detach, and *e* reinitiates. As *p* is still tethered close to the factory, it is likely to initiate again and continue the transcriptional burst. (**v**) Both polymerases have terminated, and the fiber has diffused away from the factory; both *e* and *p* enter a silent period, as both are far from the factory. (**B**) SEs increase the time *p* is close to a factory. (**i**) The structure is as Ai, but now the enhancer contains 3 promoters; as before, *p* is tethered close to a factory and likely to initiate. (**ii**) The polymerase transcribing *e* has terminated; as there are 3 SE promoters, there is a 3-fold higher chance one will collide with the factory (here *e’*) compared to A. (**iii**) *e’* has initiated, so *p* remains closely-tethered for longer and likely to initiate more often than in A. (**C**) Boundaries. (**i**) *a, b*, and *c* have initiated in different factories. (**ii**) *a* has terminated, and is more likely to visit the upper green factory compared to the distant lower one. (**iii**) *a* has re-initiated in the nearby green factory. We call *b* a boundary because it apparently prevents *a* from contacting *c*.

Our model suggests a simple mechanism for enhancer function: transcription of *e* in Figure 4Ai ensures *p* is tethered close to an appropriate factory. In other words, *e* is an enhancer of *p* because close tethering increases the probability that *p* collides with a polymerase in the factory (and so often initiates). The model also explains how enhancers can act over such great distances (Supplementary Figure S5B,C). Thus, a typical factory in a human cell is associated with ∼10 loops each with an average contour length of ∼86 kbp (Supplementary Note 1), so an enhancer anchored to it can (indirectly) tether a target promoter in any one of these other loops to the same factory. As we will see, enhancers can act over even greater distances to tether targets in a nuclear region containing an appropriate factory.

This model provides solutions to many conundrums associated with enhancers, including the following. (i) Enhancer activity depends on contact with its target promoter (71,72). We suggest the two often share a factory, and so are often in contact. (ii) Enhancers can act on two targets simultaneously, and coordinate their firing (73,74)—impossible according to classical models. In Figure 4Ai, *e* acts on both *d* and *p*, and it is easy to imagine that *d* and *p* initiate coordinately because the two polymerases involved sit side-by-side in the same factory. (iii) Promoters of protein-coding genes are often enhancers of other protein-coding genes (70,75,76). In our model, *e* is an enhancer irrespective of whether it encodes an mRNA or eRNA. (iv) Enhancers act both promiscuously and selectively. They interact with many other enhancers and targets (77–79), with ≥4 controlling a typical gene expressed during fly embryogenesis (80). At the same time, they are selective; thousands have the potential to activate a fly gene encoding an ubiquitously-expressed ribosomal-protein, whilst a different set can act on a developmentally-regulated factor (81). In our model, ‘red’ enhancers tether ‘red’ genic promoters close to ‘red’ factories, as ‘green’ ones do the same with a different set. (v) Enhancer-target contacts apparently track with the polymerase down the target (82). Thus, when mouse *Kit* becomes active, the enhancer first touches the *Kit* promoter before contacts move progressively 3′ at the speed of the pioneering polymerase. This is impossible with conventional models, but simply explained if polymerases transcribing enhancer and target are attached to one factory (Figure 4Aii,iii). (vi) Single-molecule RNA FISH shows forced looping of the β-globin enhancer to its target increases transcriptional burst frequency but not burst size (83), and this general effect is confirmed by live-cell imaging of *Drosophila* embryos (73,74). Such bursting arises because many ‘active’ genes are silent much of the time, and when active they are associated with only one elongating polymerase (Supplementary Note 8). Periods of activity do not occur randomly; rather, short bursts are interspersed by long silent periods. Bursting is usually explained by an equilibrium between ill-defined permissive and restrictive states; we explain it as follows. In Figure 4A, *p* often fires when tethered near the factory (giving a burst). Then, once *e* terminates, close tethering is lost—and *p* remains silent for as long as it remains far from an appropriate factory. RNA FISH experiments on human *SAMD4A* support this explanation; the promoter is usually silent, but adding TNFα induces successive attachments/detachments to/from a factory (44).

A related conundrum concerns how SEs work. SEs are groups of enhancers that are closely-spaced on the genetic map and often target genes determining cell identity (32,84). In Figure 4Bi, increasing the number of closely-spaced promoters (*e, e’, e’*’) in the SE increases the time *p* spends near a factory (to increase its firing probability).

### Boundaries

TAD boundaries in higher eukaryotes are often marked by CTCF; however, they are also rich in active units marked by polymerase II, nascent RNA, and factors like YY1 (4,6,22). Similarly, fly boundaries are rich in constitutively-active genes but de-enriched for insulators dCTCF and Su(Hw) (7,85). Additionally, in yeast (which lacks CTCF), boundaries are often active promoters (11). Then, does the act of transcription create a boundary? Studies in *Caulobacter crescentus*—which lacks CTCF but possesses TADs—shows it does (14). For example, in a rich medium, a rDNA gene is a strong boundary; however, this boundary disappears in a poor medium when rRNA synthesis subsides. Inserting active *rsaA* in the middle of a TAD also creates a new boundary, and boundary strength progressively falls when the length of the transcribed insert is reduced. We imagine ongoing transcription underlies boundary activity (Figure 4C).

## A GREAT MYSTERY: GENE REGULATION IS WIDELY DISTRIBUTED

Classical studies on bacterial repressors (lambda, lac) inform our thinking on how regulators work: they act locally as binary switches. We assume eukaryotes are more complicated, with more local switches, plus a few global ones (e.g. Oct3/4, Sox2, c-Myc, Klf4). We are encouraged to think this by studies on some diseases (86). For example, KLF1 regulates β globin expression by binding to its cognate site upstream of the β-globin gene (*HBB*); a C to G substitution at position -87 reduces binding, and this reduces HBB expression and causes β-thalassaemia. Therefore, we might expect binding of factors to promoters of coding genes drives phenotypic variation. However, results obtained using GWAS (genome-wide association studies)—an unbiased way of finding which genetic loci affect a phenotype—lead to a different view for many diseases; they are so unexpected that only general explanations are proffered for them (86–88).

### eQTLs

Quantitative trait loci (QTLs) are sequence variants (usually single-nucleotide changes) occurring naturally in populations that influence phenotypes. Most QTLs affecting disease do not encode transcription factors or global regulators; instead, they map to non-coding regions, especially enhancers (77,88). eQTLs are QTLs affecting transcript levels, and were also expected to encode transcription factors; but again, many do not (88,89). They also map to enhancers (88) and regulate distant genes both *cis* and *trans* (90–92). Additionally, eQTLs and their targets are often in contact (77), and one trans-eQTL can act on hundreds of genes around the genome—which often encode functionally-related proteins regulated by similar factors (88,90,92,93). In summary, eukaryotic gene regulation involves distant and distributed eQTLs that look like enhancers. Moreover, copy number of a transcript is a polygenic trait much like susceptibility to type II diabetes or human height—traits where hundreds of regulatory loci have been identified and where many more await discovery (91). This complexity is captured by the ‘omnigenic’ model, where eQTLs affect levels of target mRNAs indirectly; they modulate levels, locations, and post-translational modifications of unrelated proteins, and these changes percolate throughout the cellular network before feeding back into nuclei to affect transcription of targets (88). We suggest another—very direct—mechanism.

### A model for direct eQTL action

**Figure 5.**
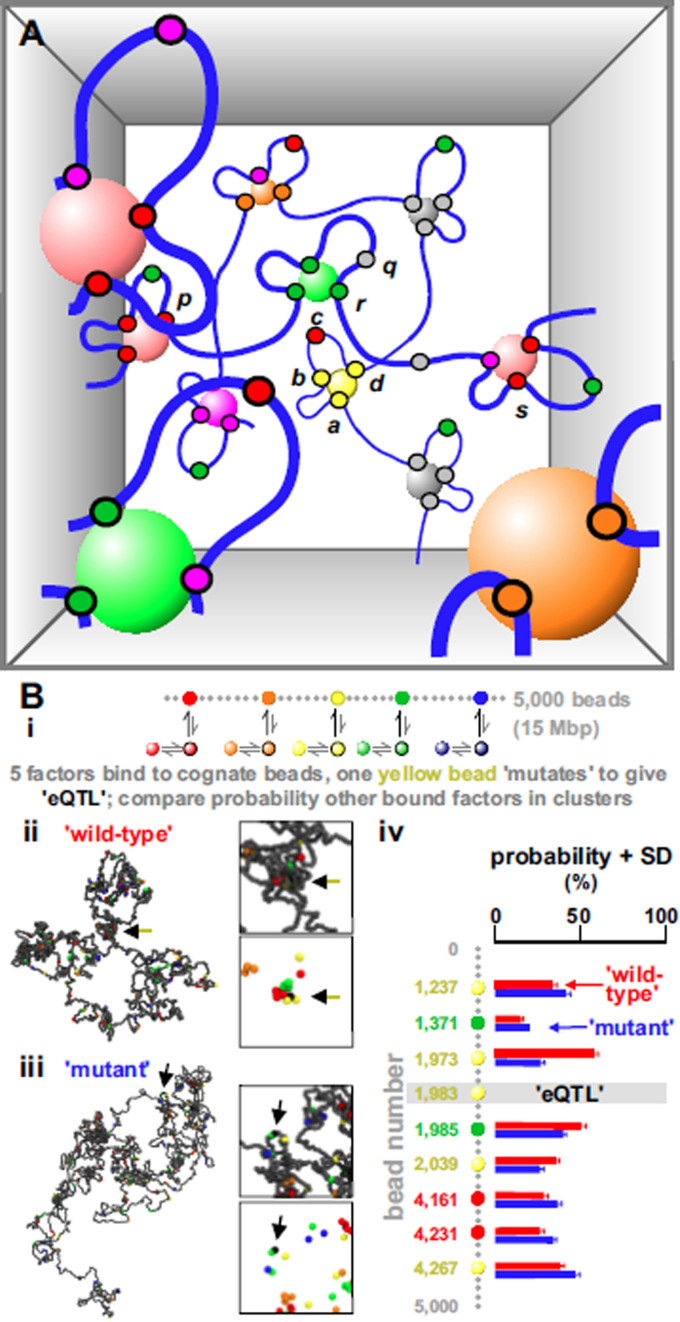
Regulation is widely distributed—an omnigenic model. (**A**) Activity of every transcription unit (small circles) in the volume depends on the activity of neighbours. *b* acts simultaneously as an enhancer of *a* and *d* (by tethering them close to the yellow factory) and a silencer of *c* (by tethering it far from a pink factory). *r* acts as a boundary between different TADs containing *p* and *s*; it also silences *q*, by preventing it from accessing a gray factory. Purple units are promiscuous, often initiating in factories of another color. (**B**) Molecular-dynamics simulations of eQTL action. (**i**) Overview. One simulation in a set of 200 involves 5 ‘factors’ (colored 30-nm spheres) binding reversibly to cognate beads of similar color randomly distributed along a ‘wild-type’ string (30-nm bead—3 kb). Factors can be ‘de-phosphorylated/phosphorylated’ to lose/gain affinity at equal rates (∼0.00001 inverse Brownian times, or ∼0.001 s^−1^). Another set involves a ‘mutant’ string with an ‘eQTL’ where yellow bead 1983 becomes non-binding. (**ii**, **iii**) Snapshots of ‘wild-type’ and ‘mutant’ fibers (bead 1983 shown black, arrowed; factors not shown). Boxes: magnifications of regions around bead 1983 with/without non-binding beads (grey). (**iv**) Positions and colors of all binding beads with altered transcription probabilities. We assume a chromatin bead is transcribed if it is within 54 nm of a factor of the corresponding color—when transcribed a bead is also typically in a cluster. Statistical significance for changes in histograms for binding beads shown is calculated assuming Gaussian statistics; histograms are different with *p*-value *p* < 0.009, and <2 beads are expected to change this much by chance.

In Figure 5A, all units in the volume determine network structure, and how often each unit visits an appropriate factory; consequently, all units directly affect production of all other transcripts. In other words, gene regulation is widely distributed. A single nucleotide change in enhancer *b* (perhaps an eQTL) might reduce binding of a ‘yellow’ factor and *b*’s firing frequency, and this has consequential effects on how often *d* and *a* are tethered close to the yellow factory—and so can initiate. But this change influences the whole network. By altering positions relative to appropriate factories, an eQTL ‘communicates’ directly with functionally-related targets, and indirectly (but still at the level of transcription) with all other genes around it in nuclear space. This neatly reconciles how eQTLs target functionally-related genes whilst having omnigenic effects (because targets often share the same specialized factory and nuclear volume, respectively).

The idea that altering one loop in a network has global effects was tested using simulations of five factors binding to cognate sites in a 5000-bead string (Figure 5Bi; Supplementary Note 6 gives details); as expected, bound factors spontaneously cluster (Figure 5Bii). We next create an ‘eQTL’ in the middle of the (‘wild-type’) string by abolishing binding to one yellow bead. This ‘mutant’ bead is now rarely in a cluster (Figure 5Biii, arrow), and it increases or decreases clustering probabilities of many other genes on the string (Figure 5Biv). As clustering determines activity, these simulations provide a physical basis for direct omnigenic effects, and open up the possibility of modeling their action. Results are robust, as, for instance, simulations with different binding affinity, or with factors and binding sites of only a single color, lead to qualitatively similar conclusions.

## LIMITATIONS OF THE MODEL

Whilst we have seen that the transcription-factory and transcription-factor models can explain many disparate observations, from phase separation of active and inactive chromatin through to eQTL action, this review would not be complete without a critical discussion of their limitations. Besides the complicated relation between TADs and transcription already reviewed, we list here some other challenges to our model.

First, the simplest version of our model does not immediately account for the bias in favor of convergent CTCF loops (over divergent ones)—which is naturally explained by the ‘loop-extrusion’ model (24,25,94,95) (see also Supplementary Note 2). However, the loop-extrusion and transcription-factor model are not alternative to one another, but complementary, so convergent loops are naturally recovered by a combined model where chromosomes are organized by both transcription factors and cohesin (bioRxiv: https://doi.org/10.1101/305359). Additionally, the motor activity behind loop extrusion, if present, may be provided by transcription itself (96) (Supplementary Note 2).

Second, the structures of mitotic and sperm chromatin pose a challenge to all models (Supplementary Notes 9 and 10). For ours, it is difficult to reconcile the persistence of loops during these stages with the common assumption that all factors are lost from chromatin. However, recent results suggest this assumption is incorrect, and that many factors do actually remain bound in mitosis (97) (Supplementary Note 9). The case of sperm is harder to explain. We speculate cohesin and other factors may still operate, and this might be sufficient to explain the observations (Supplementary Note 10).

## CONCLUSION

Seeing is believing. While clusters of RNA polymerase II tagged with GFP are seen in images of living cells (98–102), decisive experiments confirming ideas presented here will probably involve high-resolution temporal and spatial imaging of single polymerases active on specified templates. But these are demanding experiments because it is so difficult to know which kinetic population is being imaged. For example, an inactive pool of polymerase constitutes a high background; ∼80% is in a rapidly-exchanging pool, and so soluble or bound non-specifically (103). If mammalian polymerases are like bacterial ones, most at promoters fails to initiate, and—of ones that do initiate—99% abort within ∼10 nucleotides to yield transcripts too short to be seen by RNA-seq (104). Then, eukaryotic enzymes on both strands abort within 20–500 nucleotides to give products seen by RNA-seq as promoter-proximal peaks (105). On top of this, ∼60% further into genes pause for unknown periods (106). We may also think that active and inactive polymerases are easily distinguished using inhibitors, but DRB and flavopiridol do not block some polymerases at promoters (e.g. ones phosphorylated at Ser5 of the C-terminal domain), α-amanitin takes hours to act, and both α-amanitin and triptolide trigger polymerase destruction (65).

In biology, structure and function are inter-related. Here, we suggest that many individual acts of transcription determine global genome conformation, and this—in turn—feeds back to directly influence the firing of each individual transcription unit. Consequently, ‘omnigenic’ effects work both ways. [Note the term ‘omnigenic’ is used here to include both genic and non-genic transcription units.] In other words, transcription is the most ancient and basic driver of the organization in all kingdoms, with recently-evolved factors like CTCF modulating this basic structure. It also seems likely that transcription factories nucleate related ones involved in replication, repair, and recombination (40), as well as organizing mitotic chromosomes (Supplementary Note 9). They may also play important roles in other mysterious processes like meiotic chromosome pairing and transvection (107).

## Supplementary Material

Supplementary Data
