# Supplementary material for: Transcription-driven genome organization: a model for chromosome structure and the regulation of gene expression tested through simulations
Source: Nucleic Acids Res. 2018 Sep 18;46(19):9895–906. doi: 10.1093/nar/gky763 (PMC6212781; doi:10.1093/nar/gky763)
Supplement: Supplementary Data [file gky763_supplemental_files.pdf]

Transcription-driven genome organization: a model for chromosome structure and the regulation of gene expression tested through simulations: Supplementary Material

Peter R. Cook[1] and Davide Marenduzzo[2]

[1]*Sir William Dunn School of Pathology, University of Oxford, South Parks Road, Oxford, OX1 3RE*
[2]*SUPA, School of Physics, University of Edinburgh,*
Peter Guthrie Tait Road, Edinburgh, EH9 3FD, UK

---

1. Supplementary Note 1: Some properties of loops known before the invention of 3C
2. Supplementary Note 2: The "loop-extrusion" model, and other mechanisms driving enlargement of contact loops stabilized by CTCF/cohesin
3. Supplementary Note 3: Most transcription occurs in factories
4. Supplementary Note 4: Some characteristics of factories in HeLa and HUVECs
5. Supplementary Note 5: Some evidence supporting the idea that active polymerases do not track
6. Supplementary Note 6: Details of simulations.
7. Supplementary Note 7: Some additional conundrums – transcriptional interference, clustering of co-regulated genes, assembly of nuclear bodies.
8. Supplementary Note 8: Most active genes are associated with one productively-elongating polymerase
9. Supplementary Note 9: The persistence of loops during mitosis
10. Supplementary Note 10: The structure of transcriptionally-inert sperm chromatin
11. Supplementary References
12. Supplementary Fig. S1. An entropic centrifuge positions and shapes chromatin fibers.
13. Supplementary Fig. S2. Some mechanisms creating loops.
14. Supplementary Fig. S3. Cluster growth and stability seen in Brownian-dynamics simulations of chromatin.
15. Supplementary Fig. S4. Two approaches showing that active polymerases cannot track like locomotives down templates (shown as loops tethered to a sphere; pol – polymerase).
16. Supplementary Fig. S5. The development of an "NFκB" factory, and enhancer action.
17. Supplementary Fig. S6. A model and simulations indicating how promoter-factory distance can remain roughly constant despite changes in nuclear volume occurring during differentiation and evolution.

Supplementary Notes

Supplementary Note 1: Some properties of loops known before the invention of 3C

The idea that chromatin fibers are looped is an old one. Extended lampbrush loops were first described by Flemming in the 1880's (1, 2). Flemming carefully spread what we now call chromosomes of amphibian oocytes (at the stage when parental homologs pair during meiosis), and saw that most chromatin was visibly looped. In the 1970's, the genome of *Escherichia coli* – which had a circular genetic map – was also shown to be looped. Bacteria were lysed in a high salt concentration that stripped off proteins to leave naked DNA still associated with a cluster of engaged RNA polymerases (3); this DNA was supercoiled – and so looped (as supercoils are lost spontaneously from linear fibers (4)). Then, analogous experiments on human cells gave the same result; this indicated that even DNA of

organisms with linear genetic maps was looped (5). Moreover, looping and transcription were tightly correlated, as supercoils progressively disappear when transcriptionally-active chicken erythroblasts mature into inactive erythrocytes (6). Additional evidence for looping came from analyses of rates at which nucleases and γ-rays cut fibers; supercoils are released by one cut, but two nearby cuts are required to release DNA fragments from nuclei (6, 7).

Loops seen in these biochemical studies might have been generated artifactually during lysis. This provoked development of gentler methods that used "physiological" buffers and conditions where polymerases "ran-on" at rates found in vivo; then, it was likely that structure is preserved if function is also preserved. Loops under such conditions were characterized in detail, and by 1990 ($>$ 10 y before the invention of 3C) it was known that essentially all chromatin in active nuclei of men, mice, flies, and yeast was looped, and that promoters and active transcription units were major anchors (reviewed in (8)). In interphase HeLa cells, the average contour length is $\sim$ 86 kbp, with this average covering a wide range from $12.5 - 250$ kbp (9).

As discussed in the main text, improvements in Hi-C resolution allow detection of loops anchored by convergent CTCF sites (10). However, many of these loops are longer than the longest described above. Moreover, the early biochemical studies showed that loops persist during mitosis (see (9) and Supplementary Note 9); this contrasts with the failure of Hi-C to detect loops at this stage (presumably tight packing creates additional contacts that obscure ones due to looping). While Hi-C remains a powerful tool for detecting loops, it seems we must await further improvements in resolution before it is able to detect many loops in many organisms.

Supplementary Note 2: The "loop-extrusion" model, and other mechanisms driving enlargement of contact loops stabilized by CTCF/cohesin

Various mechanisms could enlarge contact loops once binding of the cohesin ring generates a small loop. We begin by noting that it remains uncertain whether cohesin stabilizes loops by acting as one ring embracing two fibers, or two connected rings each embracing one (Supplementary Fig. S2Bi; see (11)). Whatever the structure, a small loop can only enlarge if the cohesin ring (or rings) translocate down the fiber(s). This can be achieved in various ways. First, cohesin could possess an inbuilt motor (Fig. 1C); this assumption underlies the "loop-extrusion model" (12–14). This assumption is based on the fact that cohesin is an ATPase (11), and that some of its relatives are known motors (15–17). For example, SMC (structural maintenance of chromosomes) complexes may travel at $\sim$ 50 kbp/min in living bacteria (18), and yeast condensin moves $\geq$ 10 kbp mainly in one direction at $\sim$ 4 kbp/min (16). However, if a motor, cohesin would have to be more processive and faster than RNA polymerase to extrude a 1-Mbp loop in $\sim$ 25 min (its average residence time on DNA). Second, a motor like RNA polymerase could push cohesin along a fiber directly (19), or generate the supercoils that do so indirectly (20). Third, diffusion could underlie the motion (Supplementary Fig. S2B,ii; see (21)). At first glance, this seems an oxymoron – 1D diffusion gives a bi-directional random walk and not the uni-directional motion required for extrusion. However, a random walk can be biased by loading a second ring to limit movement of the first back towards the loading site; then, the second ring exerts an effective osmotic pressure that rectifies diffusion of the first. Simulations confirm this, and show that loading more rings leads to their clustering behind the pioneer. Then, if one ring in a cluster dissociates, the remainder can maintain extrusion until bound CTCF stalls it. Such molecular ratchets provide viable mechanisms driving extrusion in the required time – without invoking motors. Additionally, loop formation need not arise from unidirectional extrusion: if cohesin sticks strongly to CTCF once it finds it by diffusive sliding; this is enough to explain the formation of convergent loops (21).

As shown in Figure 2C in the main text, loop extrusion through cohesin rings in mammals seems to stall at CTCF bound to convergent cognate sites, and we would expect this to be so whether or not the CTCF is in a transcription factory. Consequently, loop extrusion and its stalling at such sites may in principle be readily accommodated within our model.

Supplementary Note 3: Most transcription occurs in factories

Some cars are assembled by enthusiasts at their own homes, but most are made in factories; are most transcripts made in factories? The answer came after permeabilizing HeLa cells in a physiological buffer (see Supplementary Note 1), labeling nascent RNA by "running-on" in biotin-CTP or Br-UTP, and immuno-labeling the resulting biotin- or Br-RNA (8, 22). Here, the challenge is to ensure that signals seen inside and outside factories accurately reflect relative amounts of transcription occurring in the two places. How can one ensure this? The answer is to run-on for longer under conditions where signal in factories grows stronger without more factories being detected (which indicates all factories are being seen), as extra-factory signal remains at background levels (indicating this signal is not due to incorporation of labelled precursors by polymerases outside factories). Quantitative light and electron microscopy (often using thin 100 nm sections to improve z-axis resolution) showed that at least 92% signal was in factories (23, 24). As experiments involving different labels, antibodies, and detection systems gave similar results, it seems that essentially all transcription occurs in factories.

Supplementary Note 4: Some characteristics of factories in HeLa and HUVECs

Factories in sub-tetraploid HeLa and diploid HUVECs are the best characterized (8). A typical nucleolar factory in HeLa (i.e., a fibrillar center or FC, plus 4 associated dense fibrillar components or DFCs) contains $\sim$ 4 rDNA templates each packed with $\sim$ 125 active molecules of

RNA polymerase I. We imagine a promoter snakes over the surface of the FC – a cluster of polymerase I and its upstream binding transcription factor, UBF. After the promoter initiates, the polymerase extrudes the promoter – which re-initiates when it reaches the next polymerase on the surface. Extruded transcripts then form the DFC. Stripping off template and transcript from the surface gives the "Christmas tree" seen in spreads (Fig. 3Ci). Finally, transcripts from one or more FCs and DFCs are assembled into ribosomes in the surrounding granular component.

The general structure of nucleoplasmic factories is like that of nucleolar ones, with nascent transcripts again found on the surface of a central core (8); now however, most active genes are productively transcribed by only one active polymerase and not the many seen on active ribosomal cistrons (see Supplementary Note 8). Thus, in a dividing HeLa cell, nascent nucleoplasmic RNA is found on the surface of a protein-rich factory core (diameter $50 - 175$ nm; mass $\sim$ 10 MDa). This core has a mass density $\sim 0.1\times$ that of a nucleosome, and so is likely to be porous. There are $\sim 6,000$ polymerase II factories per nucleus (density $\sim$ 9.3 factories/μm^3; inter-factory spacing $\sim 220 - 475$ nm), with each factory containing ~ 10 active polymerases (the remaining $\sim 80\%$ of nuclear polymerase constitutes the inactive and rapidly-exchanging soluble pool). There are also $\sim 1,200$ polymerase III factories with slightly smaller diameters. These different factories have been partially purified and their proteomes and transcriptomes analyzed; they contain the expected polymerases, associated factors, and nascent RNAs (25, 26).

In a starved HUVEC in G0 phase (which has a smaller nucleus than HeLa), there are $\sim 2,200$ polymerase II factories, and so ~ 30 in a territory occupied by a 100-Mbp chromosome. After treatment with TNFα (tumor necrosis factor α) for 30 min, there are a hundred or so specialized "NFκB" factories per nucleus (but not more than ~ 250 (27)). These numbers mean a typical gene responding to the cytokine has a good chance of visiting several "NFκB" factories every few minutes by diffusion.

Supplementary Note 5: Some evidence supporting the idea that active polymerases do not track

The extensive evidence that active polymerase do not track has been reviewed (8); three kinds are briefly summarized here. First, if active RNA polymerases track, exhaustive treatment with endonucleases should detach most DNA in a loop from tethering points; consequently, three markers of the active complex – the tracking polymerase, transcribed template, and nascent RNA – should all be detached from tethering points (Supplementary Fig. S4Ai). This experiment gave unexpected results: transcribed templates and nascent RNAs were not detached, and this pointed to active polymerases being at tethering points and so probably immobilized there (Supplementary Fig. S4Aii; see (28)). But perhaps active enzymes precipitate on to the underlying nuclear sub-structure in the unphysiological buffer used, to form new (artefactual) anchors that did not exist previously? However, using the "gentle" conditions described in Supplementary Note 1 gave the same result; removing the body of loops still did not remove any of the three markers. Instead, all remained. This again implied that active polymerizing complexes are significant tethers (29, 30), and fine-structure mapping confirmed this (31).

The second kind of evidence involved analysis of 3C contacts made between one short gene and one very long gene – 11-kbp *TNFAIP2* and 221-kbp *SAMD4A*; both genes respond to TNFα, and the short one is used as a reference point (32). Before adding TNFα, both are transcriptionally silent and rarely contact each other (both roam "outer space"; Supplementary Fig. S4B, 0 min). After adding TNFα, contacts change in a way impossible to reconcile with a model involving tracking polymerases (Supplementary Fig. S4B, $10 - 85$ min). Thus, within 10 min, the reference point (i.e., *TNFAIP2*) often contacts the *SAMD4A* promoter. After 30 min, it no longer contacts the *SAMD4A* promoter; instead, it contacts a point one-third of the way into the long gene. After 60 min, contacts shift two-thirds into *SAMD4A*, and after 85 min they reach the terminus. Such results are simply explained if polymerases active on the two genes are immobilized in one "NFκB" factory. After 10 min, both genes attach to (and initiate in) such a factory; consequently, promoter-promoter contacts are seen. As *SAMD4A* is so long, the polymerase takes 85 min before it reaches the terminus. In contrast, a polymerase on *TNFAIP2* terminates within minutes, and the short gene then goes through successive transcription cycles – sometimes attaching to (and detaching from) the same factory. If it reinitiates after 30, 60, or 85 min in the same factory (when the pioneering polymerase on *SAMD4A* has transcribed one-third, two-thirds, or all of the way along the long gene), it will contact points on *SAMD4A* that become progressively closer to the terminus – as is seen. RNA FISH coupled to super-resolution localization confirms this interpretation: intronic (nascent) RNAs copied from relevant segments of the two genes lie close enough together at appropriate times to be on the surface of one spherical factory with a diameter of $\sim$ 90 nm. Immobilization of polymerases also provides a simple explanation for the way *e-p* contacts apparently track downstream of *p* with the polymerase in Figure 4A (panels ii, iii).

The third kind of evidence involves real-time imaging of the human gene encoding cyclin D1 and its transcript as the gene becomes active (33). Thus, addition of estrogen switches on transcription in minutes, and this correlates with a reduction in the volume explored by the gene. Inhibitor studies show the constrained mobility depends on transcriptional initiation. This confirms that genes become highly confined when active.

Evidence often cited in favor of tracking polymerases comes from images of lampbrush loops. Like "Christmas trees" in "Miller" spreads (Fig. 3Ci), lampbrush loops are made by spreading a 3D structure; active polymerases and nascent RNAs (detected by immuno-labeling and autoradiography, respectively) are seen out in loops in 2D spreads (2, 34). However, transcription is required to form and maintain loops seen after spreading (35). In addition, both markers are even more concentrated in the axial chromomeres to which loops are attached (35, 36), and

no loops are seen in whole-cell sections where chromatin appears as a granular aggregate (37). As with "Christmas trees", we suggest active polymerases are stripped off factories during spreading; significantly, possible intermediates in such a process – large granular aggregates – are often seen attached to spread loops (37). Consequently, these images do not provide decisive evidence for the traditional model.

Supplementary Note 6: Details of simulations

Results in Figures 5B and Supplementary Figure S6B were obtained using Brownian dynamics (BD) simulations. These were run with the LAMMPS (Large-scale Atomic/Molecular Massively Parallel Simulator) code (38), by performing molecular dynamics simulations with a stochastic thermostat (39). Chromatin fibers are modeled as bead-and-spring polymers using FENE bonds (maximum extension 1.6 times bead diameter) and a bending potential that allows persistence length to be set (here 3 times chromatin-bead size, corresponding to a flexible polymer). Protein–protein and template–template interactions involve only steric repulsion. For template–protein interactions, we used a truncated and shifted Lennard-Jones potential (detailed below). All participants are confined within a cube with periodic boundary conditions, but strings are "unwrapped" for presentational purposes (i.e., disconnected strings are rejoined). In all cases, simulations are initialized with chromatin fibers as random walks and proteins distributed randomly with uniform density over the simulation domain. Any overlap between beads (proteins or chromatin) are eliminated with a short equilibration run with soft repulsive interactions between any two beads. Length and time scales in simulations can be mapped to physical ones, for example, by identifying bead size as 30 nm (representing 3 kbp), and a time simulation unit as 0.01 s (this unit corresponds to the square of the bead size over the diffusion coefficient of a bead in isolation; see (39, 40)).

For Figure 5B, we consider 5 different factors (red, green, blue, orange and yellow) that can bind specifically to 5 sets of cognate sites (of the same color) scattered randomly along a chromatin fiber of $5,000$ beads. The fiber represents 15 Mbp, and colored beads (cognate binding sites for factors) are spaced – on average – every 30 beads (colored beads are assigned a random color between red, green, blue, orange and yellow, with equal probability). In the set of simulations presented in Figure 5B, there are in total 172 coloured chromatin beads, of which 39 are red, 38 green, 32 blue, 33 orange and 30 yellow. The 5 factors also bind non-specifically to every other (non-colored) bead. Specific (non-specific) interaction between chromatin and protein are modeled as truncated-and-shifted Lennard-Jones potentials with interaction energy 7.1 (2.7) k_BT, with an interaction range of 54 nm. We assume factors switch between binding and non-binding states at rate $\alpha = 10^{-3}$ (41). Data presented in the histogram were averaged over 200 simulations, each of 10^5 time units. In snapshots shown, only the fiber (and only the 5 sets of cognate sites) are shown for clarity.

For Supplementary Figure S6B, we consider a single type of (non-switching) factor (so $\alpha = 0$), binding only specifically to regularly-spaced cognate sites (modeled as for Figure 5B).

For both cases, additional simulations with different interaction energy and range for DNA-protein interactions show the results to be qualitatively robust, provided that the interaction leads to multivalent binding. For Figure 5B, we have also run additional simulations with factors and binding sites of a single color, and found similar results when simulating eQTL action. Additionally, simulations with similar number of factors, but no switching give again qualitatively similar results – in this case, the protein clusters are much less dynamic as expected.

Supplementary Note 7: Some additional conundrums – transcriptional interference, clustering of co-regulated genes, assembly of nuclear bodies

In the phenomena of "transcriptional interference", firing of one promoter prevents firing of an adjacent one; this has been difficult to explain because interference extends over at least 10 kbp (42). The model and data illustrated in Figure 3D provide a simple explanation for the phenomenon. Thus, when promoter *p* is positioned anywhere in the black part of the fiber (Fig. 3Di), the fiber cannot bend back to allow *p* to reach the green volume on the surface of the factory; consequently, transcription of *e* "interferes" with (i.e., prevents) *p* from firing whilst *e* remains tethered to the factory.

In bacteria, co-regulated operons lying > 100 operons apart on the genetic map nevertheless often contact each other in 3D space (43). In man, co-functional genes are also concentrated on the genetic map and in nuclear space (44). What underlies this clustering, for which there seems to be no explanation? We suggest evolutionary pressures broadly concentrate co-regulated genes on the genetic map so they can easily access appropriate factories (Supplementary Fig. S5C).

How might functional nuclear bodies form? The nucleolus is both the prototypic factory and nuclear body. Nucleoli spontaneously assemble in human fibroblasts around tandem repeats inserted ectopically if repeats encode binding sites for UBF (the major transcription factor used by polymerase I); resulting "pseudo-nucleoli" contain UBF. If inserts also encode rDNA promoters, resulting "neo-nucleoli" contain active polymerase (45). Histone-locus bodies (HLBs) in *Drosophila* illustrate assembly of polymerase II factories. Replication-coupled histone genes are encoded by $\sim$ 100 5-kbp repeats, each with 5 histone genes, with transcription of H3 and H4 being driven by one bidirectional promoter. Ectopic insertion of 297 bp from this promoter leads to HLB assembly (46). We again suggest that the act of transcription underlies the clustering of polymerases/factors into specialized factories and the assembly of nuclear bodies – via the bridging-induced attraction (i.e., the process illustrated in Fig. 2).

Supplementary Note 8: Most active genes are associated with one productively-elongating polymerase

Many studies indicate so-called "active" genes are silent much of the time, and when active they are associated with only one productively-elongating polymerase – even in bacteria (reviewed in (47)). For example, a comprehensive survey of RNA synthesis and degradation in mouse fibroblasts shows ~ 2 mRNAs are produced per "active" gene per hour (range $\sim 0.2 - 20$ (48)). As polymerase II copies at ~ 3 kbp/min and a typical gene is ~ 30 kbp, copying occurs for only ~ 20 min in every hour – or one-third of the time. Of course, longer genes have a greater chance of being associated with > 1 polymerase (49, 50), and one rRNA gene can be transcribed simultaneously by > 100 molecules of a different polymerase – RNA polymerase I (Fig. 3C).

Supplementary Note 9: The persistence of loops during mitosis

How interphase structures change during mitosis is one of the oldest challenges in biology, and remains one today. For example, early biochemical studies showed that loops in interphase HeLa persist into mitosis without change in contour length (Supplementary Note 1; see (9)). However, no loops, TADs, or A/B compartments are seen by Hi-C in mitotic human cells (51). That loops are missed is unsurprising: resolution is insufficient against the high background induced by close packing. That A/B compartments go undetected is surprising, as Giemsa bands seen in karyotypes are such close structural counterparts (presumably they are missed because resolution is again insufficient).

The persistence of loops presents a challenge to all models – and particularly ours – as it is widely assumed that the players stabilizing loops (which might be CTCF in some models, or polymerases/factors in ours) dissociate during mitosis. Consequently, loops should disappear (as indicated by Hi-C data), or other players must take over to stabilize them (if so, what are these players?). However, recent findings suggest the underlying assumption is incorrect. Thus, many genes turn out to be transcribed during mitosis, albeit at lower levels (52), so some polymerases and factors must remain bound. Moreover, some genes and enhancers even become more active, and global levels of active marks (e.g., H3K4me2, H3K27ac) also increase (53, 54). Significantly, live-cell imaging shows that many GFP- and halo-tagged factors (e.g., Sox2, Oct4, Klf4, Foxo1/3a) – including ones previous immunofluorescence studies had shown to be lost – actually remain bound. The (apparent) loss was traced to a fixation artifact; as the fixative (paraformaldehyde) enters cells, it removes factors from the soluble pool to bias exchange with bound ones, and this strips bound molecules from chromosomes (55). Since we now know polymerases and factors do persist, they can remain the structural organizers during mitosis. In addition, they can also "bookmark" previously-active genes for future activity when chromosomes re-enter interphase (45, 46, 56, 57).

Supplementary Note 10: The structure of transcriptionally-inert sperm chromatin

The transcriptionally-inactive sperm nucleus has traditionally been viewed as a mass of unstructured and highly-compacted fibers of protamine and DNA. However, recent work on mammalian sperm shows these fibers to be far from featureless at both local and global levels. For example, their (poised) promoters and enhancers carry active marks and positioned nucleosomes reminiscent of those found in their precursors (i.e., round spermatids) and ES cells, and Hi-C analysis yields A/B compartments and TADs often defined by bound CTCF (58, 59). These findings represent a challenge for all models, and we now offer some speculations on how they might be accommodated by ours. Thus, we assume that during development of sperm, polymerases become inactive as protamines collapse pre-existing loops around factories; then, local marks, TADs, and A/B compartments would persist. Alternatively (or additionally), some polymerases might remain active as they do in mitosis (Supplemememental Note 9).

Supplementary References

1. Gall, J. G. (1996) A pictorial history: views of the cell. *Bethesda, Maryland: American Society for Cell Biology,* pp. 58–59.
2. Morgan, G. T. (2002) Lampbrush chromosomes and associated bodies: new insights into principles of nuclear structure and function. *Chromosome Res.,* **10**, 177–200.
3. Stonington, O. G. and Pettijohn, D. E. (1971) The folded genome of Escherichia coli isolated in a protein-DNA-RNA complex. *Proc. Natl. Acad. Sci. USA,* **68**, 6–9.
4. Worcel, A. and Burgi, E. (1972) On the structure of the folded chromosome of Escherichia coli. *J. Mol. Biol.,* **71**, 127–147.
5. Cook, P. and Brazell, I. (1975) Supercoils in human DNA. *J. Cell Sci.,* **19**, 261–279.
6. Cook, P. and Brazell, I. (1976) Conformational constraints in nuclear DNA. *J. Cell Sci.,* **22**, 287–302.
7. Igo-Kemenes, T. and Zachau, H. (1978) Domains in chromatin structure. In *Cold Spring Harbor symposia on quantitative biology* Cold Spring Harbor Laboratory Press Vol. 42, pp. 109–118.
8. Papantonis, A. and Cook, P. R. (2013) Transcription factories: genome organization and gene regulation. *Chemical Reviews,* **113**, 8683–8705.
9. Jackson, D., Dickinson, P., and Cook, P. (1990) The size of chromatin loops in HeLa cells. *EMBO J.,* **9**, 567–571.
10. Rao, S. S., Huntley, M. H., Durand, N. C., Stamenova, E. K., Bochkov, I. D., Robinson, J. T., Sanborn, A. L., Machol, I., Omer, A. D., Lander, E. S., et al. (2014) A 3D Map of the Human Genome at Kilobase Resolution Reveals Principles of Chromatin Looping. *Cell,* **159**, 1665 – 1680.
11. Nasmyth, K. (2011) Cohesin: a catenase with separate entry and exit gates? *Nat. Cell Biol.,* **13**, 1170 – 1177.
12. Alipour, E. and Marko, J. F. (2012) Self-organization of domain structures by DNA-loop-extruding enzymes. *Nucleic Acids Res.,* **40**, 11202–11212.
13. Sanborn, A. L., Rao, S. S. P., Huang, S.-C., Durand, N. C., Huntley, M. H., Jewett, A. I., Bochkov, I. D., Chinnappan, D., Cutkosky, A., Lia, J., et al. (2015) Chromatin extrusion

explains key features of loop and domain formation in wild-type and engineered genomes. *Proc. Natl. Acad. Sci. USA,* **112**, E6456–E6465.

14. Fudenberg, G., Imakaev, M., Lu, C., Goloborodko, A., Abdennur, N., and Mirny, L. A. (2016) Formation of Chromosomal Domains by Loop Extrusion. *Cell Rep.,* **15**, 2038–2049.
15. Eeftens, J. and Dekker, C. (2017) Catching DNA with hoops–biophysical approaches to clarify the mechanism of SMC proteins. *Nat. Struct. Mol. Biol.,* **24**, 1012 – 1020.
16. Terakawa, T., Bisht, S., Eeftens, J. M., Dekker, C., Haering, C. H., and Greene, E. C. (2017) The condensin complex is a mechanochemical motor that translocates along DNA. *Science,* **358**, 672–676.
17. Ganji, M., Shaltiel, I. A., Bisht, S., Kim, E., Kalichava, A., Haering, C. H., and Dekker, C. (2018) Real-time imaging of DNA loop extrusion by condensin. *Science,* **360**, 102–105.
18. Wang, X., Brandão, H. B., Le, T. B., Laub, M. T., and Rudner, D. Z. (2017) Bacillus subtilis SMC complexes juxtapose chromosome arms as they travel from origin to terminus. *Science,* **355**, 524–527.
19. Busslinger, G. A., Stocsits, R. R., van der Lelij, P., Axelsson, E., Tedeschi, A., Galjart, N., and Peters, J.-M. (2017) Cohesin is positioned in mammalian genomes by transcription, CTCF and Wapl. *Nature,* **544**, 503–507.
20. Racko, D., Benedetti, F., Dorier, J., and Stasiak, A. (2018) Transcription-induced supercoiling as the driving force of chromatin loop extrusion during formation of TADs in interphase chromosomes. *Nucleic Acids Res.,* **46**, 1648–1660.
21. Brackley, C. A., Johnson, J., Michieletto, D., Morozov, A. N., Nicodemi, M., Cook, P. R., and Marenduzzo, D. (2017) Non-equilibrium chromosome looping via molecular slip-links. *Phys. Rev. Lett.,* **119**, 138101.
22. Jackson, D. A., Hassan, A. B., Errington, R. J., and Cook, P. R. (1993) Visualization of focal sites of transcription within human nuclei. *EMBO J.,* **12**, 1059–1065.
23. Pombo, A., Jackson, D. A., Hollinshead, M., Wang, Z., Roeder, R. G., and Cook, P. R. (1999) Regional specialization in human nuclei: visualization of discrete sites of transcription by RNA polymerase III. *EMBO J.,* **18**, 2241–2253.
24. Faro-Trindade, I. and Cook, P. R. (2006) A conserved organization of transcription during embryonic stem cell differentiation and in cells with high C value. *Mol. Biol. Cell,* **17**, 2910–2920.
25. Melnik, S., Deng, B., Papantonis, A., Baboo, S., Carr, I. M., and Cook, P. R. (2011) The proteomes of transcription factories containing RNA polymerases I, II or III. *Nat. Methods,* **8**, 963–968.
26. Caudron-Herger, M., Cook, P. R., Rippe, K., and Papantonis, A. (2015) Dissecting the nascent human transcriptome by analysing the RNA content of transcription factories. *Nucleic Acids Res.,* **43**, e95–e95.
27. Papantonis, A., Kohro, T., Baboo, S., Larkin, J. D., Deng, B., Short, P., Tsutsumi, S., Taylor, S., Kanki, Y., Kobayashi, M., et al. (2012) TNFα signals through specialized factories where responsive coding and miRNA genes are transcribed. *EMBO J.,* **31**, 4404–4414.
28. Jackson, D., McCready, S., and Cook, P. (1981) RNA is synthesized at the nuclear cage. *Nature,* **292**, 552–555.
29. Jackson, D. and Cook, P. (1985) Transcription occurs at a nucleoskeleton. *EMBO J.,* **4**, 919–925.
30. Dickinson, P., Cook, P., and Jackson, D. (1990) Active RNA polymerase I is fixed within the nucleus of HeLa cells. *EMBO J.,* **9**, 2207–2214.
31. Jackson, D. and Cook, P. (1993) Transcriptionally active minichromosomes are attached transiently in nuclei through transcription units. *J. Cell Sci.,* **105**, 1143–1150.
32. Papantonis, A., Larkin, J. D., Wada, Y., Ohta, Y., Ihara, S., Kodama, T., and Cook, P. R. (2010) Active RNA polymerases: mobile or immobile molecular machines? *PLoS Biol.,* **8**, e1000419.
33. Germier, T., Kocanova, S., Walther, N., Bancaud, A., Shaban, H. A., Sellou, H., Politi, A. Z., Ellenberg, J., Gallardo, F., and Bystricky, K. (2017) Real-Time Imaging of a Single Gene Reveals Transcription-Initiated Local Confinement. *Biophys. J.,* **113**, 1383–1394.
34. Gall, J. G. and Nizami, Z. F. (2016) Isolation of Giant Lampbrush Chromosomes from Living Oocytes of Frogs and Salamanders. *J. Vis. Exp.,* e54103.
35. Gall, J. G. and Murphy, C. (1998) Assembly of lampbrush chromosomes from sperm chromatin. *Mol. Biol. Cell,* **9**, 733–747.
36. Snow, M. and Callan, H. (1969) Evidence for a polarized movement of the lateral loops of newt lampbrush chromosomes during oogenesis. *J. Cell Sci.,* **5**, 1–25.
37. Mott, M. and Callen, H. (1975) An electron-microscope study of the lampbrush chromosomes of the newt Triturus cristatus. *J. Cell Sci.,* **17**, 241–261.
38. Plimpton, S. (1995) Fast Parallel Algorithms for Short-Range Molecular Dynamics. *J. Comp. Phys.,* **117**, 1–19.
39. Brackley, C. A., Johnson, J., Kelly, S., Cook, P. R., and Marenduzzo, D. (2016) Simulated binding of transcription factors to active and inactive regions folds human chromosomes into loops, rosettes and topological domains. *Nucleic Acids Res.,* **44**, 3503–3512.
40. Michieletto, D., Chiang, M., Coli, D., Papantonis, A., Orlandini, E., Cook, P. R., and Marenduzzo, D. (2017) Shaping epigenetic memory via genomic bookmarking. *Nucleic Acids Res.,* **46**, 83–93.
41. Brackley, C. A., Liebchen, B., Michieletto, D., Mouvet, F. L., Cook, P. R., and Marenduzzo, D. (2017) Ephemeral protein binding to DNA shapes stable nuclear bodies and chromatin domains. *Biophys. J.,* **28**, 1085–1093.
42. Emerman, M. and Temin, H. M. (1986) Quantitative analysis of gene suppression in integrated retrovirus vectors. *Mol. Cell Biol.,* **6**, 792–800.
43. Xie, T., Fu, L.-Y., Yang, Q.-Y., Xiong, H., Xu, H., Ma, B.-G., and Zhang, H.-Y. (2015) Spatial features for Escherichia coli genome organization. *BMC Genomics,* **16**, 37.
44. Thévenin, A., Ein-Dor, L., Ozery-Flato, M., and Shamir, R. (2014) Functional gene groups are concentrated within chromosomes, among chromosomes and in the nuclear space of the human genome. *Nucleic Acids Res.,* **42**, 9854–9861.
45. Grob, A. and McStay, B. (2014) Construction of synthetic nucleoli and what it tells us about propagation of sub-nuclear domains through cell division. *Cell cycle,* **13**, 2501–2508.
46. Salzler, H. R., Tatomer, D. C., Malek, P. Y., McDaniel, S. L., Orlando, A. N., Marzluff, W. F., and Duronio, R. J. (2013) A sequence in the Drosophila H3-H4 Promoter triggers histone locus body assembly and biosynthesis of replication-coupled histone mRNAs. *Dev. Cell,* **24**, 623–634.
47. Finan, K. and Cook, P. R. (2011) Transcriptional initiation: frequency, bursting, and transcription factories. *Genome Organization and Function in the Cell Nucleus,* pp. 235–254.
48. Schwanhäusser, B., Busse, D., Li, N., Dittmar, G., Schuchhardt, J., Wolf, J., Chen, W., and Selbach, M. (2011) Global quantification of mammalian gene expression control. *Nature,* **473**, 337–342.
49. Larkin, J. D., Cook, P. R., and Papantonis, A. (2012) Dynamic reconfiguration of long human genes during one transcription cycle. *Mol. Cell. Biol.,* **32**, 2738–2747.
50. Larkin, J. D., Papantonis, A., Cook, P. R., and Marenduzzo, D. (2013) Space exploration by the promoter of a long human

gene during one transcription cycle. *Nucleic Acids Res.,* **41**, 2216–2227.

51. Naumova, N., Imakaev, M., Fudenberg, G., Zhan, Y., Lajoie, B. R., Mirny, L. A., and Dekker, J. (2013) Organization of the mitotic chromosome.. *Science,* **342**, 948–53.
52. Palozola, K. C., Donahue, G., Liu, H., Grant, G. R., Becker, J. S., Cote, A., Yu, H., Raj, A., and Zaret, K. S. (2017) Mitotic transcription and waves of gene reactivation during mitotic exit. *Science,* **358**, 119–122.
53. Liang, K., Woodfin, A. R., Slaughter, B. D., Unruh, J. R., Box, A. C., Rickels, R. A., Gao, X., Haug, J. S., Jaspersen, S. L., and Shilatifard, A. (2015) Mitotic transcriptional activation: clearance of actively engaged Pol II via transcriptional elongation control in mitosis. *Mol. Cell,* **60**, 435–445.
54. Liu, Y., Chen, S., Wang, S., Soares, F., Fischer, M., Meng, F., Du, Z., Lin, C., Meyer, C., DeCaprio, J. A., et al. (2017) Transcriptional landscape of the human cell cycle. *Proc. Natl. Acad. Sci. USA,* **114**, 3473–3478.
55. Teves, S. S., An, L., Hansen, A. S., Xie, L., Darzacq, X., and Tjian, R. (2016) A dynamic mode of mitotic bookmarking by transcription factors. *Elife,* **5**, 1–24.
56. Grob, A., Colleran, C., and McStay, B. (2014) Construction of synthetic nucleoli in human cells reveals how a major functional nuclear domain is formed and propagated through cell division. *Genes Dev.,* **28**, 220–230.
57. Hsiung, C. C.-S. and Blobel, G. A. (2016) A new bookmark of the mitotic genome in embryonic stem cells. *Nat. Cell Biol.,* **18**, 1124–1125.
58. Battulin, N., Fishman, V. S., Mazur, A. M., Pomaznoy, M., Khabarova, A. A., Afonnikov, D. A., Prokhortchouk, E. B., and Serov, O. L. (2015) Comparison of the three-dimensional organization of sperm and fibroblast genomes using the Hi-C approach. *Genome Biol.,* **16**(1), 77.
59. Jung, Y. H., Sauria, M. E., Lyu, X., Cheema, M. S., Ausio, J., Taylor, J., and Corces, V. G. (2017) Chromatin states in mouse sperm correlate with embryonic and adult regulatory landscapes. *Cell Rep.,* **18**(6), 1366–1382.
60. Cook, P. R. and Marenduzzo, D. (2009) Entropic organization of interphase chromosomes. *J. Cell. Biol.,* **186**, 825–834.
61. Donev, A., Cisse, I., Sachs, D., Variano, E. A., Stillinger, F. H., Connelly, R., Torquato, S., and Chaikin, P. M. (2004) Improving the density of jammed disordered packings using ellipsoids. *Science,* **303**, 990–993.
62. Man, W., Donev, A., Stillinger, F. H., Sullivan, M. T., Russel, W. B., Heeger, D., Inati, S., Torquato, S., and Chaikin, P. (2005) Experiments on random packings of ellipsoids. *Phys. Rev. Lett.,* **94**, 198001.
63. Hart, J. C. (1994) Distance to an ellipsoid. *Graphics gems IV,* pp. 113–119.
64. Stevens, T. J., Lando, D., Basu, S., Atkinson, L. P., Cao, Y., Lee, S. F., Leeb, M., Wohlfahrt, K. J., Boucher, W., O'Shaughnessy-Kirwan, et al. (2017) 3D structures of individual mammalian genomes studied by single-cell Hi-C. *Nature,* **544**, 59–64.
65. Wang, Y., Nagarajan, M., Uhler, C., and Shivashankar, G. (2017) Orientation and repositioning of chromosomes correlate with cell geometry–dependent gene expression. *Mol. Biol. Cell,* **28**, 1997–2009.
66. Khalil, A., Grant, J., Caddle, L., Atzema, E., Mills, K., and Arnéodo, A. (2007) Chromosome territories have a highly nonspherical morphology and nonrandom positioning. *Chromosome Res.,* **15**, 899–916.
67. Hnisz, D., Shrinivas, K., Young, R. A., Chakraborty, A. K., and Sharp, P. A. (2017) A phase separation model for transcriptional control. *Cell,* **169**, 13–23.
68. Larson, A. G., Elnatan, D., Keenen, M. M., Trnka, M. J., Johnston, J. B., Burlingame, A. L., Agard, D. A., Redding, S., and Narlikar, G. J. (2017) Liquid droplet formation by HP1α suggests a role for phase separation in heterochromatin. *Nature,* **547**, 236–240.
69. Strom, A. R., Emelyanov, A. V., Mir, M., Fyodorov, D. V., Darzacq, X., and Karpen, G. H. (2017) Phase separation drives heterochromatin domain formation. *Nature,* **547**, 241–245.
70. Bon, M., Marenduzzo, D., and Cook, P. R. (2006) Modeling a self-avoiding chromatin loop: relation to the packing problem, action-at-a-distance, and nuclear context. *Structure,* **14**, 197–204.

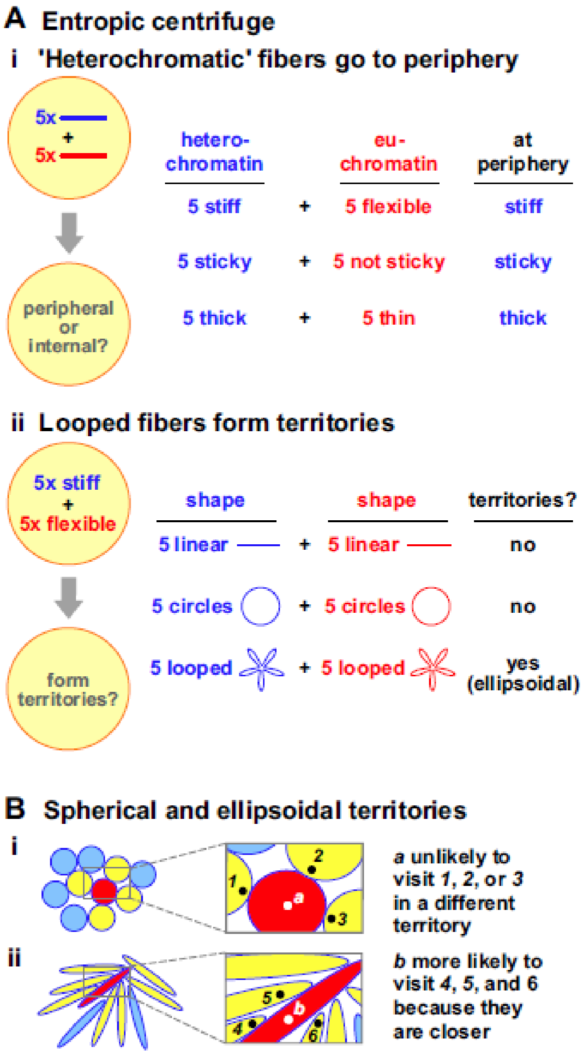

Figure S1: An entropic centrifuge positions and shapes chromatin fibers. **A.** Monte Carlo simulations involved two sets of 5 fibers "diffusing" in a sphere, and determination of ultimate positions and shapes (60). **(i)** "Heterochromatic" v "euchromatic" sets; heterochromatic fibers with higher stiffness, stickiness for others of the same type, and thickness tend to end up at the periphery. **(ii)** Stiff v flexible sets (linear, circular, or looped); only looped fibers form territories (others intermingle). **B.** Ten ellipsoids ("territories") pack together more tightly than 10 spheres of similar volume, and may contact more neighbors; they are also less likely to become locally jammed because they have one thinner axis and so can escape through smaller gaps (61, 62). For example, consider an ellipsoidal territory (principle axes 1 : 2.9 : 4.5) and a spherical one of similar volume (diameter 4 μm). Then, 22% of the ellipsoidal volume is within 125 nm of the surface compared to 18% of the spherical one, and the average shortest path of any point in the ellipsoid to the surface is 300 nm (i.e., 60% of the shortest path in the sphere; calculated as described in (63)). Ellipsoidal territories are found in haploid mouse embryonic stem (ES) cells (64), NIH 3T3 cells (principle axes 1 : 2 : 3.5 or 1 : 1.6 : 2.3 depending on substrate (65)), and pro-B nuclei (principle axes 1 : 2.9 : 4.5 (66)). **(i)** The red sphere touches 4 yellow ones. **(ii)** The red ellipsoid touches 7 yellow ones, and *b* at its center is closer to *4*, *5*, and *6* than *a* is to *1*, *2*, and *3*.

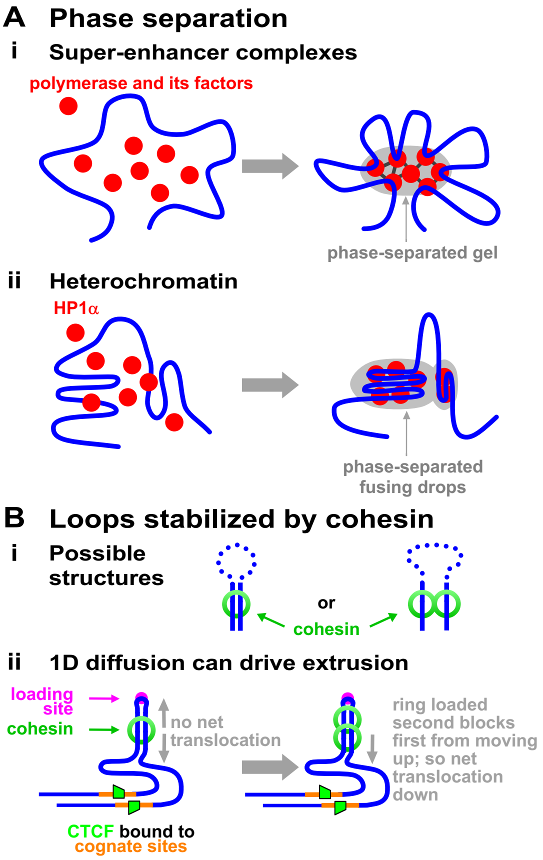

Figure S2: Some mechanisms creating loops. **A.** Phase separation. **(i)** Super-enhancer complexes (67). The polymerase and its factors bind to promoters and form a phase-separated cluster or gel stabilized by multivalent interactions (black lines); this cluster/gel organizes surrounding loops. This structure is essentially the same as that of a transcription factory. **(ii)** Heterochromatin. HP1α forms (phase-separated) liquid-like drops if local concentrations are high enough; it staples fibers together into compact structures with mini-loops (68, 69). Here, two liquid drops have just fused to compact two heterochromatic regions. **B.** Stabilizing loops with cohesin, and enlarging them by 1D diffusion. **(i)** Two possible arrangements for a loop stabilized by cohesin; we assume here that one cohesin ring embraces two duplexes (left), but the same argument applies if two rings each embrace one duplex (right). **(ii)** A loop stabilized by cohesin could enlarge by 1D diffusion as follows. After binding to the loading site, cohesin then diffuses in a 1D random walk along the fiber; consequently, there is no net translocation along the fiber, and the loop does not enlarge. However, this random walk is biased if a second ring loads at the same loading site, as the second now limits movement of the first back towards the loading site. In practice, the second exerts an effective osmotic pressure that rectifies diffusion of the first. This molecular ratchet provides a viable mechanism driving extrusion without the need to invoke a motor.

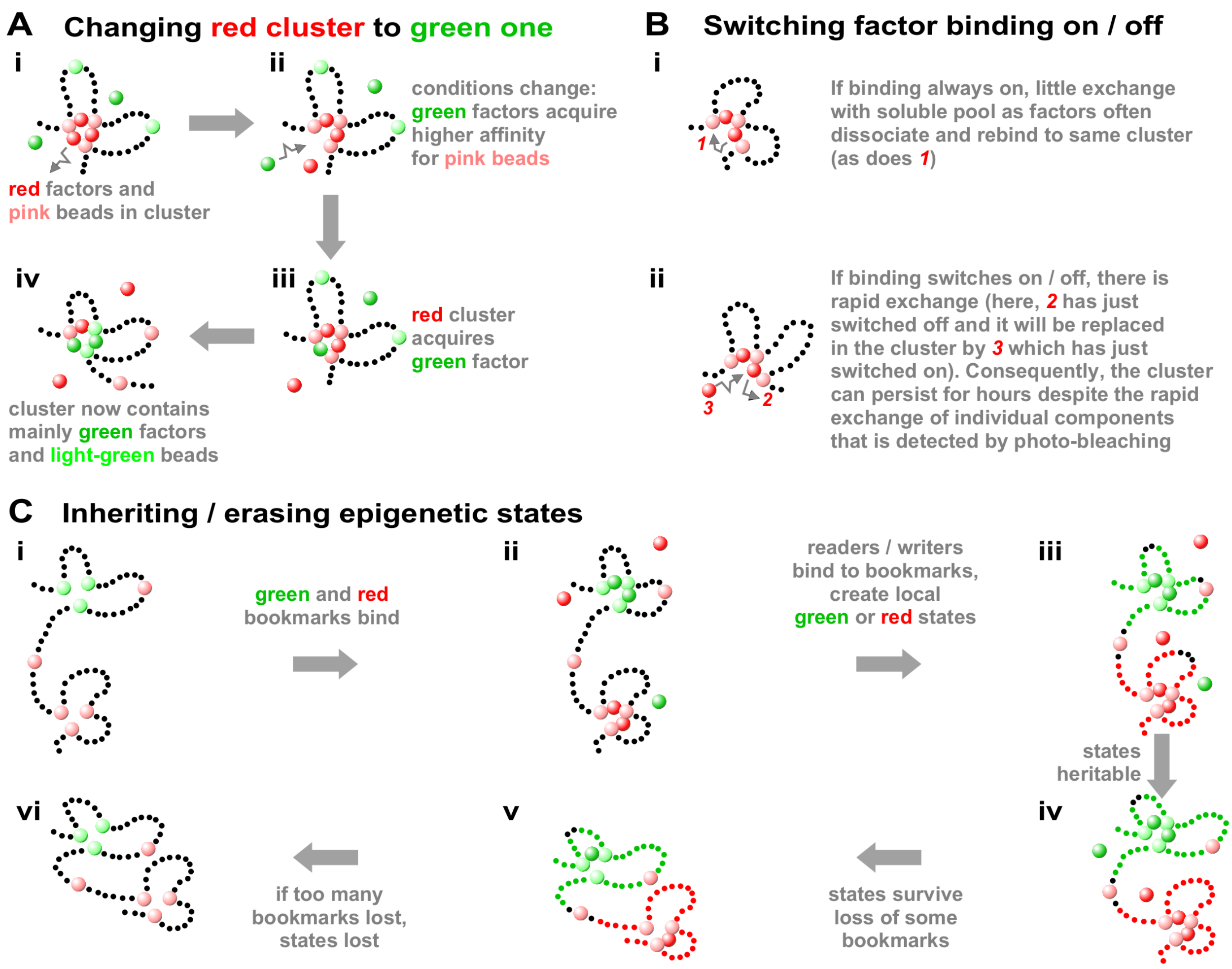

Figure S3: Cluster growth and stability seen in Brownian-dynamics simulations of chromatin. **A.** Cluster "differentiation"; pink/light-green beads represent genes expressed before/after differentiation (39). **(i)** Different factors (green, red spheres) bind to cognate sites (light-green, pink beads). Initially, green factors have no affinity for any bead, but red factors can bind to pink ones; red clusters form (as Fig. 2A,B). Here, a red factor is about to dissociate (arrow). **(ii)** Green factors are phosphorylated; their affinity for pink beads is now higher than that of red factors. **(iii)** A green factor has replaced a red one in the cluster due to higher-affinity binding. **(iv)** More green factors replace red ones in the cluster due to their higher affinity (see also Fig. 2B). **B.** Switching binding on/off by "phosphorylation"/"dephosphorylation" facilitates exchange with the soluble pool, as seen experimentally in photo-bleaching experiments (41). **(i)** If factors exist permanently in a binding state, high local concentrations ensure they dissociate and rebind to the same cluster (as *1*); consequently, there is little exchange with the soluble pool. **(ii)** If factors switch between binding/non-binding states, they often exchange (here, the cluster loses *2* and gains *3*) and clusters can persist for hours as constituents exchange in seconds (as seen experimentally). **C.** Inheriting and erasing epigenetic states (40). **(i)** A naïve string lacking "epigenetic marks". **(ii)** Green and red "bookmarks" (e.g., factors related to active and inactive chromatin) bind to cognate beads to form green and red clusters (as Fig. 2A,B). **(iii)** Bookmarks now recruit epigenetic "readers" and "writers" (not shown) that "mark" histones in nearby beads (colored dots in the string). **(iv)** Resulting "epigenetic states" and "epigenetic domains" persist through continued action of readers/writers. **(v)** The system quickly restores marks when either marks or bookmarking factors are removed randomly (mimicking losses occurring during "semi-conservative replication" or "mitosis"). **(vi)** States are lost as the concentration of bookmarks becomes too dilute to maintain them (or if the genomic sequence binding the bookmark is excised, not shown (40)).

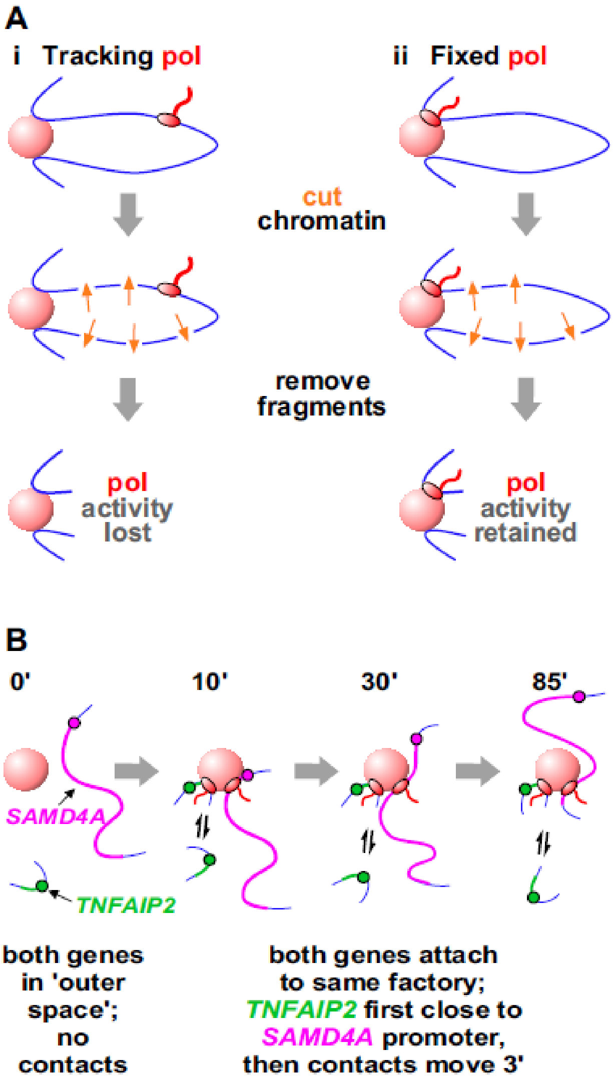

Figure S4: Two approaches showing that active polymerases cannot track like locomotives down templates (shown as loops tethered to a sphere; pol – polymerase). **A.** The experiment involves nuclease digestion, removal of resulting fragments, and detection of 3 markers – remaining nascent RNA, transcribed sequence, and polymerizing activity (oval). **(i)** If the active polymerase tracks, cutting chromatin should separate it from anchor points; when small fragments of chromatin are now removed, all 3 markers will be lost. **(ii)** If the polymerase anchors the loop, cutting chromatin and removing fragments will leave all 3 markers; this is the result seen. **B.** Analysis of 3C contacts made between 11-kbp *TNFAIP2* and 221-kbp *SAMD4A*. Before adding TNFα (0 min) both genes are silent and not in contact. Ten minutes after adding TNFα, both genes become active; *TNFAIP2* now often contacts the *SAMD4A* promoter (but not downstream segments). After 30 min, *TNFAIP2* no longer contacts the *SAMD4A* promoter; instead, it contacts a point one-third into *SAMD4A*. After 60 min, contacts shift two-thirds into *SAMD4A*, and by 85 min they reach the terminus. These results are impossible to explain if polymerases track, but easily explained if the two active polymerases are immobilized in one factory. Then, after 10 min, both genes attach to (and initiate in) this factory (giving promoter-promoter contacts). As it takes 85 min to transcribe *SAMD4A* whilst *TNFAIP2* is transcribed in minutes, the short gene goes through successive transcription cycles by attaching to (and detaching from) the factory. Consequently, whenever *TNFAIP2* is transcribed, it will lie close to the point on *SAMD4A* that is being transcribed at that moment.

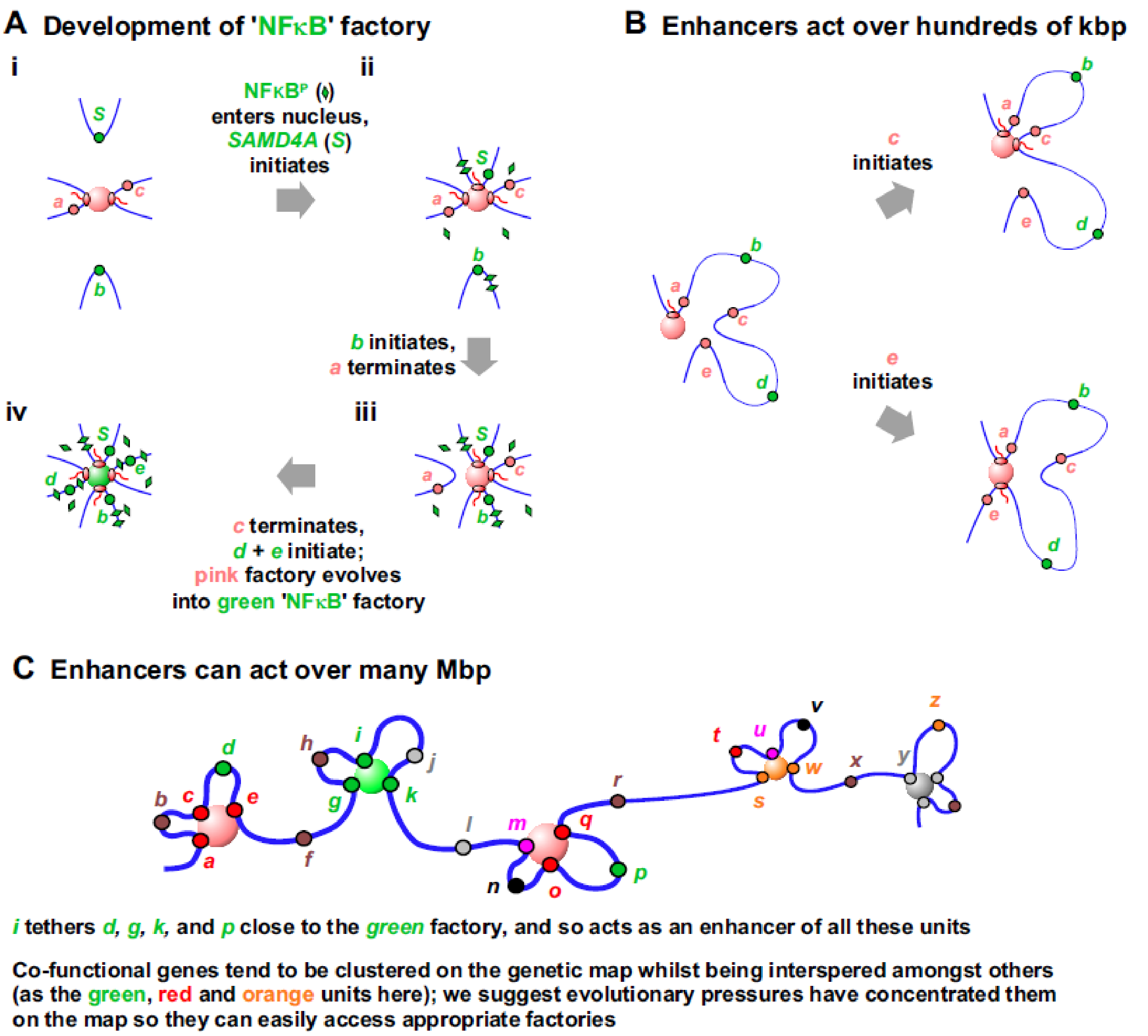

Figure S5: The development of an "NFκB" factory, and enhancer action, in a human cell. **A.** Development of an "NFκB" factory on addition of TNFα (27). **(i)** Before adding TNFα. Promoters a and c have initiated in the pink factory; S (*SAMD4A*) and b may visit the factory, but they cannot initiate as the required transcription factor is absent. 3C shows that S and b rarely contact each other. **(ii)** 10 min after adding TNFα. NFκB is now phosphorylated (NFκBP), it entered the nucleus, and – when S now visits the factory – it initiates. S encodes many NFκB-binding sites, and exchange of NFκBP from these sites now creates a local concentration of the factor in/around the factory. **(iii)** b visits the factory and initiates. 3C shows S and b now often contact each other. Both genes encode NFκB-binding sites, so the local concentration of the factor in/around the factory increases. **(iv)** The pink factory develops into a (green) "NFκB" factory specializing in transcribing green units as other green promoters initiate. **B.** Enhancers can act over hundreds of kbp. Initially, a, c, and e were transcribed in the factory, but c and e have just terminated. a still tethers c and e close to the factory, and so both are likely to re-initiate. Consequently, a is an enhancer of c and e. As $\sim$ 10 loops of $\sim$ 86 kbp are typically anchored to one human factory, a can tether genes lying $\sim$ 860 kbp away near the factory, and so enhance activity. **C.** Enhancers can act over many Mbp. About 4 Mbp of a human chromosomes are shown (again, only some of the $\sim$ 10 loops/factory are shown). Transcription units a – z tend to be transcribed in factories of the same color, except for purple ones that are promiscuous. Imagine i is transcribed often. Consequently, d, g, k and p will be tethered near the green factory so i acts as their enhancer (even though some lie $>$ 1 Mbp away). Co-functional genes (i.e., ones with promoters of similar color) also tend to be clustered on the genetic map, as shown here; we suggest this is the result of evolutionary pressures ensuring they can easily access appropriate factories. Note that green promoters are interspersed amongst pink ones, so it is possible this structure evolves into one where all green promoters are simultaneously transcribed in one green factory (while all pink promoters are transiently silent), and then into another structure where all pink promoters are transcribed in one pink factory (while all green promoters remain silent).

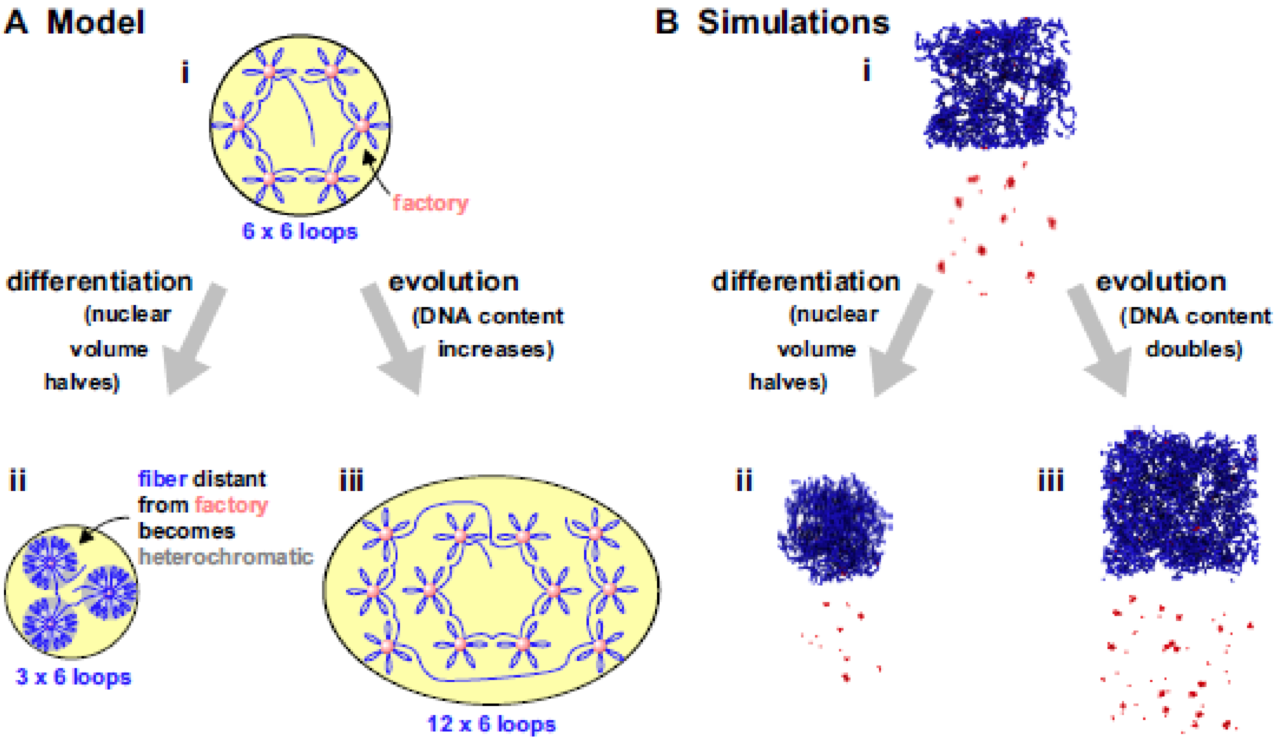

Figure S6: A model and simulations indicating how promoter-factory distance can remain roughly constant despite changes in nuclear volume occurring during differentiation and evolution. **A.** Model (24). **(i)** All nucleoplasmic chromatin of a mouse ES cell is represented by one chromatin fiber organized into 6 loops around 6 factories. **(ii)** Differentiation into a cell with half the nucleoplasmic volume. Experimental data show the smaller nucleus has half the number of factories and active polymerases, but a similar factory diameter and density (i.e., number of factories per unit nucleoplasmic volume). As half the number of polymerases are active but the total amount of DNA is similar, our model requires the total number of loops should fall and contour length increase. Therefore, one might expect the volume of chromatin around each factory to increase. However, two factors probably combine to ensure it does not. First, polymer physics indicates that as loop length doubles, the radius of the volume occupied increases only $\sim$ 1.5-fold (70). Second, the fiber distant from a factory probably becomes heterochromatic and so more tightly packed (grey zone). Consequently, increased loop length has little effect on factory density. In other words, the system self-regulates so the average gene remains just as far away from a factory despite the volume change. **(iii)** Changes occurring during evolution as DNA content increases 2-fold (original data involved comparison of a mouse ES cell and a newt cell with 10-fold more DNA). Factory diameter and density remain constant, as nucleoplasmic volume and total number of active polymerases increase. As there is more DNA and more polymerases are active, we suggest loop contour-length remains constant; the system again self-regulates. **B.** Snapshots from 3 Brownian-dynamics simulations consistent with the model in (A). Simulations (details in Supplementary Note 6) involve a string ("chromatin" fiber) of blue beads (each representing 3 kbp) diffusing in a cube as red spheres ("factors"/"polymerases") bind reversibly to cognate beads spaced every 90 kbp along the string (also shown blue, interaction energy and range – 7.1 kBT and 54 nm). Upper and lower panels show images of all beads plus "factors", or just "factors", in the cube at the end. **(i)** Stem cell (15-Mbp fiber, 100 factors, 1.5 μm side cube). Bound red beads spontaneously cluster (as Fig. 2A). **(ii)** During "differentiation", the same amount of chromatin is confined in half the volume (1.2 μm side cube), and there are half the number of factors and binding beads (reflecting silencing of half binding sites). The number of red beads/cluster, cluster density, and cluster diameter are as (i), but cluster number halves. **(iii)** During "evolution" to a cell with twice the DNA, "fiber" length doubles to 30 Mbp, but "chromatin" and "factor" density remain constant (1.89 μm side cube; 200 "factors"). Cluster number doubles, but the number of red beads/cluster, cluster density, and cluster diameter are again as (i).
